# Regenerable Membrane Sensors for Ultrasensitive Nanoplastic Quantification Enabled by A Data-driven Raman Spectral Processing Algorithm

**DOI:** 10.1021/acs.est.5c05396

**Published:** 2025-07-29

**Authors:** Ziyan Wu, Sarah E. Janssen, Michael T. Tate, Mohan Qin, Haoran Wei

**Affiliations:** † Department of Civil and Environmental Engineering, 5228 University of Wisconsin−Madison, Madison, Wisconsin 53706, United States; ‡ Upper Midwest Water Science Center, U.S. Geological Survey, Madison, Wisconsin 53726, United States; § Environmental Chemistry and Technology Program, University of Wisconsin–Madison, Madison, Wisconsin 53706, United States

**Keywords:** nanoplastics, membrane sensor, Raman microspectroscopy, freshwater, data processing algorithm

## Abstract

The detection of nanoplastics (NPs) in complex natural water systems is hindered by matrix interferences and limitations in current analytical techniques. This study presents Pre_seg, a Raman spectral processing algorithm integrated with regenerable anodic aluminum oxide (AAO) membrane sensors, for ultrasensitive, rapid, and quantitative NP detection at the single-particle level. The AAO membranes function as both filtration substrates and Raman sensors, reducing sample loss and contamination. Pre_seg incorporates statistically determined thresholds for signal-to-noise ratios (SNRs) and full width at half maximums (fwhms) across segmented spectral ranges, effectively minimizing noise and enhancing accuracy and sensitivity of NP detection. Pre_seg achieved 93.5% prediction accuracy of NPs and ≥90.4% rejection accuracy for non-NP entries. Mixed NPs were quantified at the lowest concentration of 0.5 μg L^–1^. The robustness of Pre_seg was validated in eutrophic and oligotrophic lake matrices following oxidation digestion pretreatment to mitigate organic interferences. Furthermore, the AAO membrane sensors demonstrated stability through multiple regeneration and reuse cycles. This innovative approach advances NP detection by enabling scalable, customizable, and environmentally relevant monitoring.

## Graphical abstract

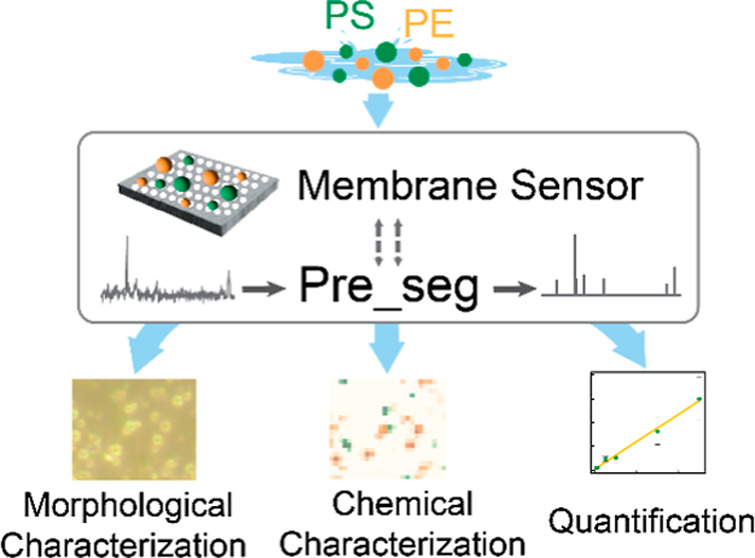

## 1 Introduction

Since the discovery of small plastic debris in marine environments in the 1970s,^1–3^ research on various aspects of these materials, including their distribution, fate, and ecological impacts, has surged in aquatic systems.^4,5^ Small plastic debris, categorized as microplastics (MPs) (1–5000 μm) and nanoplastics (NPs, <1 μm), have emerged as a critical environmental contaminant.^6,7^ Compared to the larger counterparts, NPs exhibit higher toxicity and bioavailability, thus posing significant risks to aquatic organisms.^8,9^ Once ingested, NPs can aggregate in the gut, penetrate tissues and cells, and disrupt cellular functions, threatening species such as algae, sea urchin, and mussels.^10^ Despite their harmful effects, the occurrence of NPs in natural water systems remained poorly understood,^11^ with only limited studies analyzing NPs in real environmental samples. NPs, including polystyrene (PS), polypropylene (PP), polyethylene (PE), polyethylene terephthalate (PET), and polyvinyl chloride (PVC), have been detected in seawater,^12^ coastal sediments,^13^ snow,^14^ and lakes.^15^ Concerns regarding the presence of NPs in natural water systems, particularly freshwater systems, have intensified in recent years due to their critical role as sources of drinking water.^7,15^ However, our understanding of NPs in these systems is still at the infant stage, largely due to the lack of reliable detection methods.^5,7^

Simultaneously imaging, quantifying, and characterizing NPs in water at the single-particle level remains a significant challenge.^7,16^ Mass spectrometry (MS)-based techniques and vibrational spectroscopic tools have been employed for NP detection in water samples.^12,17–19^ However, MS-based techniques, such as pyrolysis gas chromatography–mass spectrometry (py-GC/MS), primarily provide bulk characterization, capturing the overall properties of the NPs but lacking single-particle resolution. To address this limitation, additional imaging instruments are often required to complement MS-based techniques for morphological analysis of individual NPs.^7,20^ Alternatively, vibrational spectroscopic tools, including surface-enhanced Raman spectroscopy (SERS) and stimulated Raman scattering (SRS) microscopy, have been used to image and chemically characterize NPs in water samples.^12,18,19,21,22^ While these techniques offer detection limits as low as the μg L^–1^ level, previous studies have typically focused on either chemical characterization or imaging of NPs,^19,22,23^ rather than achieving all three goals—quantification, characterization, and imaging—simultaneously. Furthermore, single-particle detection of NPs remains time-consuming with existing analytical methods due to the prolonged scanning time required by the vibrational spectroscopic tools to capture their small dimensions.^19^ There is a pressing need for sensing tools that can achieve high-quality, single-particle detection of NPs with reduced time and labor input.

Existing analytical methods to investigate the morphology, abundance, or chemical composition of NPs have predominantly been demonstrated in pure or relatively simple water matrices.^12,19^ However, the impact of complex freshwater matrices on the performance and accuracy of these methods remains underexplored. For instance, natural organic matter (NOM) concentrations in freshwater systems can vary by more than 1 order of magnitude across different environments.^24,25^ Such variations may introduce differing levels of background interference with analysis using vibrational spectroscopic tools, potentially obscuring NP signals.^26^ A range of spectral processing algorithms have been developed to differentiate MPs from background interference, including algebra-based and logic-based statistical methods^27^ and machine learning-assisted approaches such as classification^28^ and regression models.^29^ However, the effectiveness of these algorithms for NP analysis remains uncertain, as smaller NPs produce weaker signals and are more susceptible to interference from water matrix and substrate background.^30^ To date, only a few spectral algorithms, including random forest^31^ and principal component analysis (PCA),^32^ have been employed for NP imaging or characterization. There is still a lack of spectral processing algorithms specifically designed to mitigate matrix and background-induced interference for the quantitative detection of NPs in real environmental matrices.^5,14^

To address these challenges, we developed a Raman spectral processing algorithm integrated with a membrane sensor to reliably separate and quantitatively detect NPs at the single-particle level in waters from the Great Lakes Basin. PS and PE NPs were selected as model particles due to their well-defined morphology, narrow size distribution, commercial availability, and ubiquitous presence in freshwater environments.^8,17^ Anodic aluminum oxide (AAO) membranes—selected for their negligible Raman response—served both to recover NPs from water samples and to perform in situ quantification and chemical characterization via Raman spectroscopy. A Raman spectral processing algorithm, Pre_seg, was developed to extract quantitative information of NPs from noisy, interference-prone Raman spectra. By applying statistically determined thresholds for signal-to-noise ratio (SNR) and full-width at half-maximum (FWHM) of the target NPs, this algorithm effectively filters out interferent signals, greatly improving detection sensitivity. Both single-sized and mixed-sized NPs were quantified using Raman maps generated by Pre_seg. The regeneration capacity of the AAO membrane sensors was validated across multiple cycles, confirming their reusability. The reliability of the developed detection approach was further validated in eutrophic and oligotrophic water matrices from the Great Lakes Basin, highlighting the significance of sample pretreatment for accurate NP analysis in real environmental water matrices. This study introduces a reliable and customizable Raman spectral processing algorithm tailored for nanoplastic quantification, effectively transforming AAO membranes into a sustainable sensing tool for rapid, accurate, and ultrasensitive NP detection at environmentally relevant levels in complex environmental matrices.

## 2 Materials and Methods

### 2.1 Materials

Anodic aluminum oxide (AAO) membranes (pore sizes of 20 and 200 nm) with a diameter of 25 mm were purchased from Whatman (Maidstone, UK). These pore sizes were selected based on their commercial availability. Two suspensions containing single-size PS nanospheres with average sizes of 300 and 500 nm were purchased from Phosphorex (Hopkinton, MA). PE nanosphere powders of size ranging from 200 to 9900 nm were purchased from Cospheric (Moorpark, CA). PS and PE NPs were selected as model NPs to evaluate and validate the performance of the developed method due to their well-defined morphology and size distribution, commercial availability, and ubiquitous presence in the freshwater environments.^14,33–35^

### 2.2 AAO Membrane Sensor Characterization

An optical contact angle goniometer (OCA 15 plus, Dataphysics, NC) was used to investigate the hydrophilicity of the AAO membrane sensor by measuring the contact angle of a water droplet on the membrane surface. Surface morphology of the AAO membrane sensors was assessed with a scanning electron microscope (SEM) (Zeiss GeminiSEM 450, Zeiss, Germany). A confocal Raman spectrometer (Horiba XploRA PLUS) was used to collect Raman spectra from the pristine AAO membrane sensor under 532 nm laser excitation with an acquisition time of 0.1 s. A data set containing approximately 1100 spectra of pristine AAO membranes was created as one of the three non-NP spectra data sets to test the rejection accuracy of Pre_seg.

### 2.3 Membrane Filtration of NPs

A dead-end membrane filtration system was used to recover NPs from water samples. The system consisted of a glass holder, glass funnel, glass Erlenmeyer flask, and a stainless-steel clamp. Water samples spiked with NPs were filtered through the AAO membrane sensor. After filtration, the AAO membrane sensors were air-dried before Raman analysis. All membrane sensors have been rinsed with deionized (DI) water before use to eliminate potential interferences.^36^

### 2.4 Dark-Field and Raman Imaging of NPs

A confocal Raman spectrometer (Horiba XploRA PLUS) with dark-field imaging function was used to visualize and analyze NPs on 20 nm AAO membrane sensors. Dark-field images of PS and PE NPs were captured using a 100× objective, allowing detailed observation of their distribution and morphology on the membrane surface. After dark-field imaging, Raman maps were acquired under 532 nm laser excitation. For each Raman map, the Raman spectra of individual pixels within the imaging area were measured with an acquisition time of 0.1 s.

### 2.5 Procedures of Pre_seg

Pre_seg was developed using Python to eliminate spectral noise and reduce the dimensionality of individual Raman spectra. The full algorithm consists of four key steps, i.e., baseline correction, peak detection, denoising based on signal-to-noise ratio, and denoising based on FWHM. These steps collectively refined Raman spectra by considering Raman shift, band intensity, and bandwidth, generating low-dimensional representations of PS and PE NP Raman spectra. The distribution of the band widths of five characteristic Raman bands of PS and PE were calculated based on 50 spectra, which was used to establish the FWHM thresholds in the last step of Pre_seg. Based on the observed bandwidth distribution of standard NPs and MPs, Raman spectra were segmented into two ranges (i.e., 300–2750 cm^–1^ and 2750–3500 cm^–1^) for targeted analysis of NPs.

### 2.6 Quantification of PS NPs

Suspensions of 500 nm PS NPs with concentrations of 1, 5, 10, 40, 100 μg L^–1^ and 300 nm PS NPs with concentrations of 0.5, 1, 5, 10, 30, and 50 μg L^–1^ were prepared by diluting the purchased stock suspension. A 100 mL aliquot of each suspension was filtered through the 20 nm AAO membrane sensor for analysis. Raman maps were acquired over an area of 22 × 26 pixels under 532 nm laser excitation. The pixel areas for Raman mapping were 500 × 500 and 300 × 300 nm^2^ for 500 and 300 nm PS NPs, respectively. For each concentration, the intensity of the dominant PS Raman band at 996 cm^–1^ was calculated and averaged across all pixels in three independent Raman maps. The averaged Raman map intensity was then plotted against the nominal concentrations provided by the manufacturer to generate calibration curves for single-sized PS NPs of 300 and 500 nm.

Three sets of generalized calibration curves for mixed-sized PS NPs were produced. To produce the first generalized calibration curve, the calibration curve of single-sized PS NPs of 300 nm was scaled up to match the scale of the calibration curve of 500 nm PS NPs based on the Raman map area ratio between the two types of NPs. To create the second generalized calibration curve, the calibration curve of single-sized PS NPs of 500 nm was scaled down to match the scale of the calibration curve of 300 nm PS NPs based on the Raman map area ratio between these two types of NPs. The third generalized calibration curve was produced by averaging the first and second generalized calibration curves. Mixed suspensions of 300 and 500 nm PS NPs with total concentrations of 2 and 20 μg L^–1^ were prepared to test the accuracy of the generalized calibration curves. In total, six Raman maps in an imaging area of 22 × 26 pixels were acquired under 532 nm laser excitation with step sizes of 500 and 300 nm.

### 2.7 AAO Membrane Sensor Regeneration

Different regeneration methods were explored in this study, including the traditional methods for ceramic membrane cleaning used in wastewater treatment plants and the baking method. Traditional methods for ceramic membrane cleaning in wastewater treatment plants involve multiple steps, including acid cleaning, backwashing, and disinfection.^37,38^ We designed a cleaning cycle involving backwashing and disinfection to regenerate the 200 nm AAO membrane sensor. The 200 nm AAO membranes were chosen over the 20 nm membranes due to their higher water flow rates, which reduced the time required for the filtration experiments. While this method effectively removed membrane fouling and restored water flux, residue impurities were still observed on the membrane surface after a single cleaning cycle (Figure S1).

Alternatively, the baking method was tested to regenerate the AAO membrane sensors. A 100 mL aliquot of 500 nm PS NPs with a concentration of 0.05 mg L^–1^ was filtered through the pristine 200 nm AAO membrane sensor. After filtration, the membrane sensor was baked at 500 °C in a muffle furnace for 2 h. The filtration and baking process constituted one regeneration cycle. After the first cycle, the regenerated membrane sensor was analyzed using a confocal Raman spectrometer to investigate the surface change and cleanness. The regeneration cycle was repeated four more times, with surface analysis conducted after each cycle. Finally, a 100 mL suspension of 500 nm PS NPs at 0.05 mg L^–1^ was filtered through the regenerated AAO membrane sensor. The membrane sensors with PS NPs were then analyzed to verify the performance of the regenerated 200 nm AAO membrane for NP detection.

### 2.8 Detecting NPs in Lake Water

Lake Mendota water was sampled from the lakeshore in the City of Madison, WI in August 2024. Lake Michigan water was sampled from the lakeshore in the City of Evanston, IL in November 2024. The sampled water from both lakes was stored at 4 °C before use. A TOC analyzer (TOC-L, Shimadzu Corporation, Columbia, MD) was used to measure dissolved organic carbon (DOC). PE NPs (in the size range of 200 to 9900 nm) and 500 nm PS NPs were spiked into the lake water samples for further analysis. PE NPs were spiked into all lake water at a concentration of 5 mg L^–1^, while PS NPs were spiked into Lake Mendota and Lake Michigan at concentrations of 10 and 20 μg L^–1^, respectively.

Oxidative digestion was applied to the raw lake water from Lake Mendota and the spiked samples from Lake Mendota and Lake Michigan. A 30% hydrogen peroxide solution was used to digest and clean the lake water samples by heating them to 60 °C for 12 h. The digested samples were first filtered through a 1 μm polycarbonate track-etch (PCTE) membrane to remove larger particles. The filtrate was then filtered through 20 nm AAO membrane sensors, which were subsequently analyzed using Raman spectroscopy. A data set of approximately 1100 spectra from AAO membrane sensors after filtering digested Lake Mendota water was created as one of the three non-NP spectra data sets to test the rejection accuracy of Pre_seg.

To investigate how the interferences from raw lake water affect NP imaging and detection on the AAO membrane sensors, raw lake water from Lake Mendota and spiked raw Lake Mendota water containing 500 nm PS and PE NPs were filtered through 1 μm PCTE membranes and subsequently through 20 nm AAO membrane sensors. The AAO membrane sensors with raw (undigested) samples were then analyzed using Raman spectroscopy. A data set of approximately 1100 spectra from AAO membrane sensors after filtering raw Lake Mendota water was generated as one of the three non-NP spectra data sets to test the rejection accuracy of Pre_seg.

## 3 Results and Discussion

### 3.1 Chemical Characterization of NPs at the Single-Particle Level

To evaluate their suitability for NP detection, the hydrophobicity and surface structure of AAO membranes were characterized prior to analysis. The membranes exhibited hydrophilic properties (Figure 1a), with SEM images revealing a porous and flat surface structure (Figures 1b,c, S2, Text S1). The flat, homogeneous surface of AAO membrane sensors ensures uniform distribution and high-quality imaging of individual NPs recovered via a dead-end filtration system (Figure 1d), paving the way for quantitative detection of NPs of various sizes in later sections. Raman spectra from more than 50 individual PS NPs (300 and 500 nm) on AAO membranes were highly reproducible, displaying consistent band positions and intensities with an ultrashort collection time of 0.1 s (Figure 1e,i), providing a solid foundation for reliable Raman mapping and accurate detection of NPs on AAO membrane sensors.

**Figure 1:**
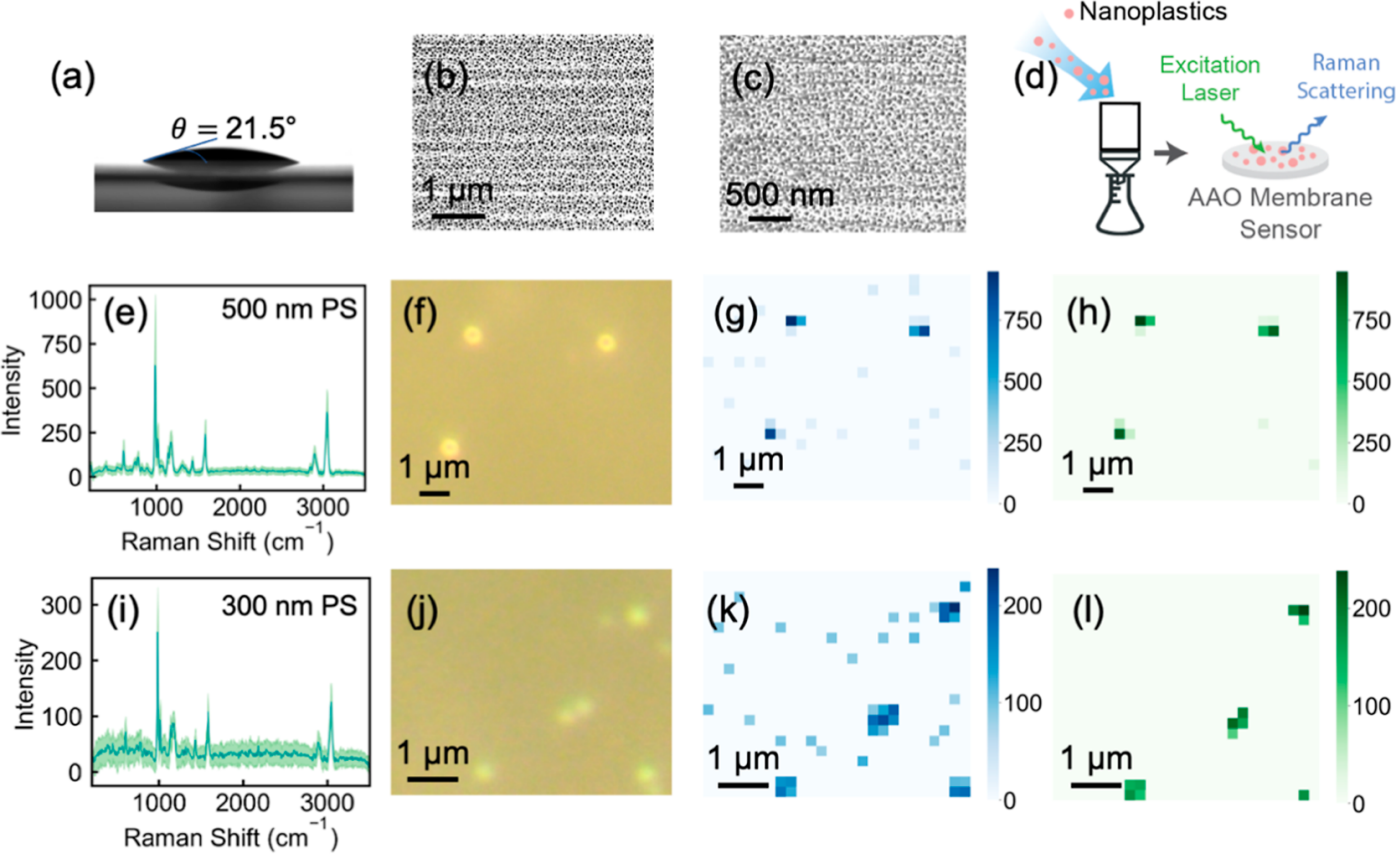
Characterization of anodic aluminum oxide (AAO) membranes and Raman analysis of NPs on AAO membranes. (a) Contact angle of a deionized water droplet on a pristine AAO membrane (pore size = 20 nm). (b,c) SEM images of a 20 nm AAO membrane at different magnifications. (d) Schematic diagram of NP collection and detection on AAO membranes using a dead-end membrane filtration setup to separate 300 and 500 nm PS NPs from water. Retained NPs were directly imaged and analyzed via Raman spectroscopy. Raman spectra of (e) 500 and (i) 300 nm PS NPs on 20 nm AAO membranes, with individual spectra from 50 particles measured and averaged (dark green line); the lighter green shading represents one standard deviation. Dark-field images of (f) 500 and (j) 300 nm PS NPs on 20 nm AAO membranes. Raman maps of (g) 500 and (k) 300 nm PS NPs processed with Pre_fun. Raman maps of (h) 500 and (l) 300 nm PS NPs processed with Pre_seg. Raman maps were generated by tracking the PS Raman band at 996 cm^–1^. Each Raman spectrum was collected with an integration time of 0.1 s for one accumulation. Scanning step sizes were 500 and 300 nm for (g,h) and (k,l), respectively. The concentrations of 500 and 300 nm PS were 1 μg L^–1^.

In our previous study, we developed a Raman spectral processing algorithm, Pre_fun, for low-micrometer microplastic (LMMP) detection.^39^ While Pre_fun successfully generated Raman maps delineating individual 500 and 300 nm NPs,^39^ significant background interference was observed, particularly for smaller NPs (300 nm) or at environmentally relevant concentrations (Figures 1f,g,j,k, S3 and S4). Background signals from the pristine AAO membrane interfered more prominently with 300 nm NPs than with 500 nm NPs due to their lower band intensity (∼200 CCD counts), which was only slightly higher than that of the pristine AAO membranes (Figures S5 and S6). At environmentally relevant concentrations (e.g., 1 and 0.5 μg L^–1^),^14,35^ the mapping areas exhibited a markedly increased background-to-NP pixel ratio relative to higher concentrations, generating a substantial amount of “false positive” signals that compromised the analysis of both 500 and 300 nm NPs (Figures 1g,k, S3 and S4).

Background interference from AAO membranes not only obscured the visual interpretation of PS NP distribution but also complicated the statistical quantification of PS NPs. To address this challenge, we developed Pre_seg, a data-driven Raman spectral processing algorithm designed to remove background interference while preserving the distinctive Raman signatures of target NPs, enabling precise and potentially automated detection. After being processed with Pre_seg, the Raman maps of 500 and 300 nm PS NPs exhibited a clean background and accurately delineated the distribution of NPs on the AAO membranes across all tested concentrations (0.5–100 μg L^–1^) (Figures 1h,l, S3 and S4). By effectively mitigating background interference (false positive signals), Pre_seg transformed the AAO membrane into a functional membrane sensor for the quantitative detection of NPs. The detailed methodology and functionality of Pre_seg are elaborated in the following sections.

### 3.2 Pre_seg Tailored for Raman Spectral Processing

Unlike conventional Raman spectral processing approaches, Pre_seg utilizes a streamlined four-step process: baseline correction (BC), peak detection (P), denoising based on signal-to-noise ratio (S), and further denoising using full width at half-maximum (FWHM-s). This approach produces a low-dimensional Raman spectrum that effectively preserves targeted NP Raman bands while eliminating spectral noises. To evaluate the performance of Pre_seg, we experimentally generated a data set comprising Raman spectra from individual 500 nm PS NPs on AAO membranes, pristine AAO membranes, AAO membranes after digested lake water filtration, and AAO membranes after raw lake water filtration.

Pre_seg effectively eliminated spectral noises and generated low-dimensional PS NP spectra with consistent Raman band patterns across the data set (Figures 2 and S7). The first step, BC, removed background signals, primarily fluorescence, without reducing spectral dimensionality (Figure S8). The second step, P, compressed the spectra by filtering out 70% of noise entries (Figure 2a). The third and fourth steps, S and FWHM-s, further compressed the PS NP Raman spectra to less than 10 dimensions (Figures 2b,c and S7), significantly lowering data storage requirements and computation costs for NP detection. Dominant Raman bands of PS NPs (996, 1027, 1597, 2905, and 3053 cm^–1^) were preserved, while noise was effectively removed from the processed spectra (Figures 2c and S7). Pre_seg also effectively denoised the Raman spectra from blank AAO membranes, mitigating interference from substrate background with PS NP analysis (Figures 2d–f and S9).

**Figure 2:**
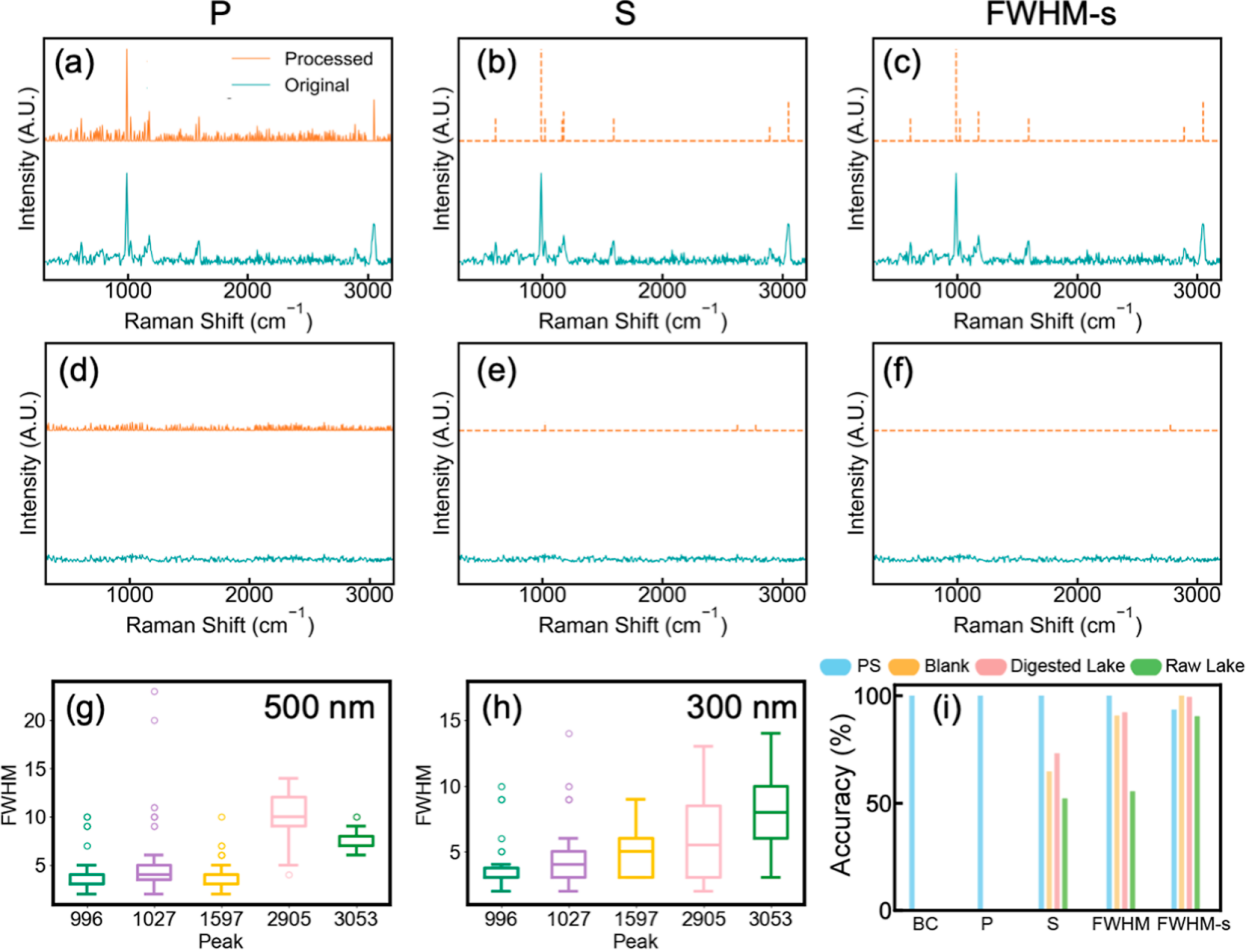
Procedure and performance of Pre_seg for improving PS NP analysis on 20 nm AAO membranes. Raman spectra of 500 nm PS NPs on a 20 nm AAO membrane after (a) peak detection, (b) denoising based on signal-to-noise ratio (SNR), and (c) denoising based on full width at half-maximum (FWHM). Raman spectra of a pristine 20 nm AAO membrane after (d) peak detection, (e) SNR-based denoising, and (f) FWHM-based denoising. Processed and unprocessed Raman spectra (both baseline-corrected) are plotted as orange and teal lines, respectively. Calculated FWHM values for five characteristic Raman bands of (g) 500 and (h) 300 nm PS NPs based on 50 spectra. (i) Prediction and rejection accuracy of Pre_seg at different processing stages for 500 nm PS NPs (PS, 200 spectra), blank AAO membranes (blank, 1100 spectra), AAO membranes after raw lake water filtration (raw lake, 1100 spectra), and AAO membranes after digested lake water filtration (digested lake, 1100 spectra). BC stands for baseline correction. P stands for peak detection. S stands for denoising based on SNR. FWHM stands for denoising based on FWHM using a unified threshold. FWHM-s stands for denoising based on FWHM using two thresholds applied to different spectral segments. Prediction and rejection accuracies for each data set were evaluated based on the detection of a Raman band around 996 cm^–1^. Details on accuracy calculations are further documented in Text S2.

Pre_seg required data-driven parameter optimization to effectively eliminate spectral noise and reduce spectral dimensions for NP detection. Each Raman band in a Raman spectrum corresponds to a vibrational mode of a chemical bond,^40^ characterized by its Raman shift, intensity, and width.^41,42^ Pre_seg incorporated all three parameters in its analysis. In the P step, relevant Raman shifts were identified by locating local intensity maxima. The S step refined peak selection by only retaining peaks that exceeded the noise level by at least two standard deviations. The SNR of two standard deviations were chosen based on iterative testing with PS Raman spectra. Finally, the FWHM-s step used bandwidth as a filter to isolate Raman bands specific to the target NPs. The FWHM of the dominant PS Raman bands followed a consistent trend (Figure 2g,h). Bands within 300–2750 cm^–1^ were relatively narrow (bandwidth: 2–10 cm^–1^, median: 4 cm^–1^), whereas those in the 2750–3500 cm^–1^ range were broader (bandwidth: 5–15 cm^–1^, median: >10 cm^–1^). The carbon–hydrogen backbone of common nanoplastics produces closely spaced Raman shifts within the 2750–3500 cm^–1^ range,^43,44^ often leading to overlapping bands and increased bandwidth. The same trend was observed in the Raman spectra of standard PE NPs (Figure S10) and pristine and environmentally eroded microplastics (PS, PE, PVC, and PP) from two open-access microplastic libraries (Figure S11).^45,46^

These statistically determined band widths laid out the foundation for the last step of Pre_seg. Raman spectra of PS NPs were segmented into two ranges: 300–2750 and 2750–3500 cm^–1^. After the S step, Raman bands within each range were further refined based on the experimentally determined median FWHM. Notably, this segmentation step substantially improved the rejection accuracy of non-PS NP spectra (i.e., from pristine membranes and membranes after lake water filtration) by up to 62.9% (Figure 2i). The previously developed algorithm for LMMPs, Pre_fun, achieved 100% accuracy in detecting PS NPs, but only 52.2% accuracy of rejecting non-NP spectra from membranes after raw lake water filtration due to organic interference (Figure S12). In contrast, spectral segmentation in Pre_seg enhanced the differentiation of PS NP spectra from non-PS spectra, particularly in raw lake water, underscoring its role in effectively reducing false positives for accurate NP detection (Figure 2i). Each step in Pre_seg progressively improved the rejection accuracy of non-PS spectra (Figure 2i) and minimized background interference in Raman maps for both 500 and 300 nm PS NPs (Figure S13) with a minor decrease in the prediction accuracy of PS NPs. Overall, Pre_seg achieved 93.5% prediction accuracy for PS NPs, and over 90.4% rejection accuracy for non-PS spectra from pristine AAO membrane and AAO membranes filtered with digested and raw lake water (Figure 2i). This high rejection rate of non-NP signals provides a clean Raman map background for robust NP quantification, as detailed in the following section.

### 3.3 Quantification of NPs Enabled by Pre_seg

PS NPs were successfully quantified on AAO membrane sensors with exceptional sensitivity for both 500 and 300 nm PS NPs (Figure 3), owing to Pre_seg’s ability to eliminate background noise across Raman maps. Without Pre_seg, background inference not only distorted PS NP signals in the Raman maps (Figures S3 and S4), but also reduced detection sensitivity, particularly at environmentally relevant concentrations (Figure S14). By removing background interference, Pre_seg produced Raman maps with accurate PS NP signals and clean backgrounds, enabling linear calibration curves and precise quantification for both 500 and 300 nm PS (Figure 3a–d). Following Pre_seg processing, the averaged Raman intensities at the lowest tested concentrations −1 μg L^–1^ for 500 nm NPs and 0.5 μg L^–1^ for 300 nm NPs—were 29-fold and 14-fold of the averaged intensities of the blank controls, respectively. This underscores the critical role of background interference reduction in achieving reliable and ultrasensitive NP quantification.

**Figure 3:**
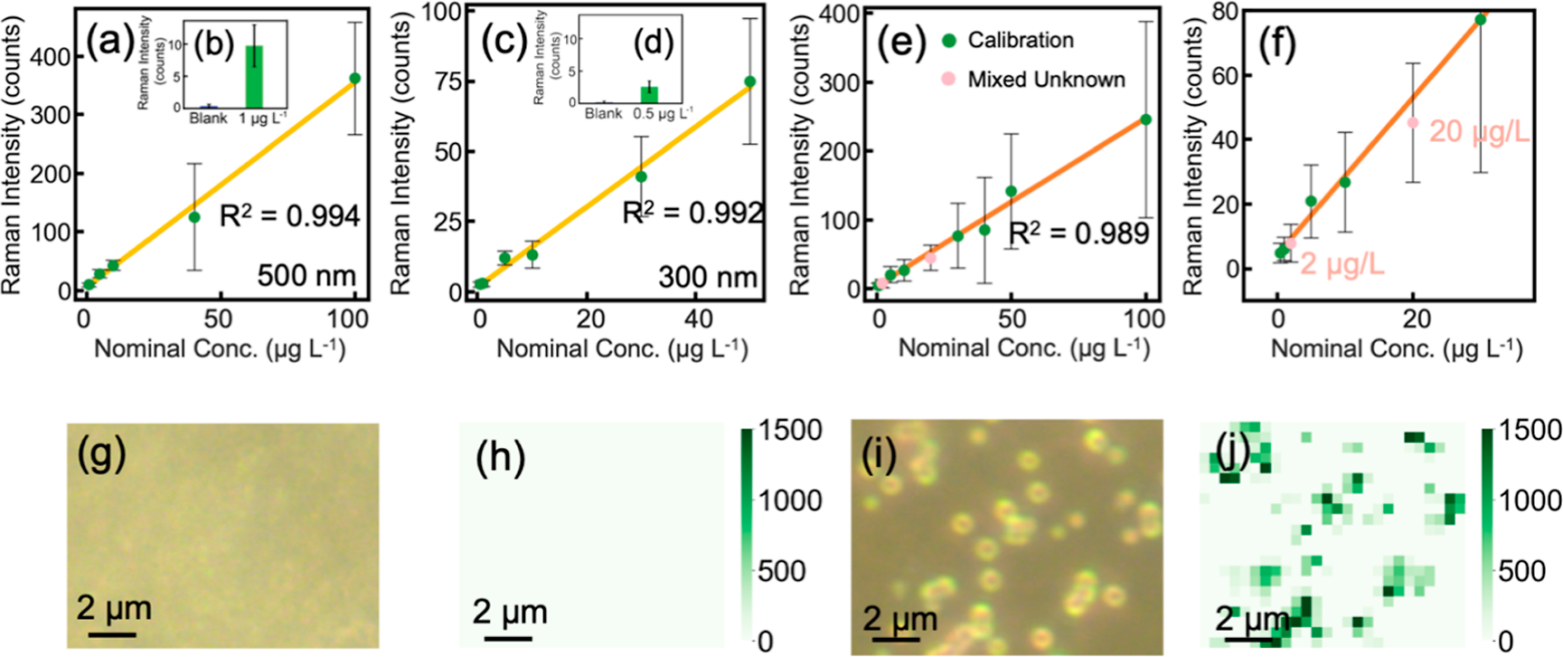
Quantitative analysis of 500 and 300 nm PS NPs on 20 nm AAO membranes, and dark-field images and Raman maps of 500 nm PS on regenerated AAO membranes. (a) Calibration curve for quantitative analysis of 500 nm PS NPs on 20 nm AAO membranes. (b) Averaged Raman intensities of Raman maps tracking the 996 cm^–1^ band for a blank sample (blue, left bar) and a 1 μg L^–1^ sample of 500 nm PS NPs (green, right bar) on 20 nm AAO membranes. (c) Calibration curve for quantitative analysis of 300 nm PS NPs on 20 nm AAO membranes. (d) Averaged Raman intensities of Raman maps tracking the 996 cm^–1^ band for a blank sample (blue, left bar) and a 0.5 μg L^–1^ sample of 300 nm PS NPs (green, right bar) on 20 nm AAO membranes. (e) Calibration curve for mixed 500 and 300 nm PS NPs on 20 nm AAO membranes, where green dots represent single-sized 500 or 300 nm PS NPs, and pink dots represent mixed samples. (f) Zoomed-in view of (e). (g) Dark-field image of an AAO membrane after five regeneration cycles without respiking NPs. (h) Corresponding Raman map of the regenerated AAO membrane processed with Pre_seg. (i) Dark-field image of an AAO membrane after five regeneration cycles with respiked 500 nm PS NPs. (j) Corresponding Raman map of the regenerated AAO membrane with respiked PS NPs processed with Pre_seg. Raman maps were generated by tracking the PS Raman band at 996 cm^–1^ with a step size of 500 nm. The integration time for each measurement was 0.1 s.

Pre_seg also enabled accurate characterization and quantification of mixed-size PS NPs on AAO membrane sensors. Quantifying mixed submicro NPs at μg L^–1^ levels has been extremely challenging in previous studies.^8,17^ In this study, Raman maps on AAO membrane sensors precisely identified mixed 500 and 300 nm PS NPs on the membrane sensors (Figure S15). However, background interference initially hindered accurate NP quantification. Unprocessed Raman maps produced nonlinear calibration curves when combining data from 500 and 300 nm NPs (Figure S16), whereas Pre_seg processing yielded strong linear calibration curves (Figures 3e,f and S17). The spatial resolution of Raman maps, determined by the laser scanning step size, played a pivotal role in NP quantification. Lower resolutions are likely to miss some smaller PS NPs, while higher resolutions overestimated larger ones. Consequently, mixed PS NPs were underpredicted when calibration curves were based on the spatial resolution of larger NPs (step size: 500 nm) and overpredicted when based on smaller NPs (step size: 300 nm) (Figure S17). To address these discrepancies, an average calibration curve was acquired by integrating spatial resolution effects from both scanning step sizes. The generation of the average calibration curve was detailed in the Method section. This generalized calibration curve accurately predicted the concentrations of mixed 500 and 300 nm PS NPs at 2 and 20 μg L^–1^ (Figure 3e,f), highlighting the significance of spatial resolution in developing a generalized NP quantification method.

### 3.4 Regeneration of AAO Membranes for NP Detection

To assess the stability of the AAO membrane sensor for repeated use, its detection performance for NPs was investigated after multiple regeneration and reuse cycles. The used membrane sensor surface was completely cleaned without visible changes after one and five regeneration cycles by simply heating in air (see Methods for details). This was confirmed by clean dark-field images and Raman maps after the regeneration of used AAO membranes (Figures S18a,b and 3g,h). Dark-field images of 500 nm PS NPs on regenerated AAO membrane sensors confirmed the uncompromised imaging capability (Figures 3i and S18c). Additionally, Raman maps of 500 nm PS NPs on the regenerated AAO membranes maintained high accuracy and clean background, comparable to those of unused membrane sensors (Figures 3j and S18d). These results collectively demonstrated the reusability of the AAO membrane sensor, highlighting its potential as a reliable and inexpensive tool for accurate NP detection through multiple regeneration and reuse cycles.

### 3.5 Detecting NPs in Natural Lake Water

Sample digestion played an essential role in the reliable detection of NPs in environmental water. Chemical compounds from raw lake water caused blurry dark-field images (Figure 4a,c), hindering the visualization of PS and PE NPs on the membrane sensors. Raman spectra of the membrane sensors after filtering Lake Mendota water (Figure S19a,c), a eutrophic lake located in the Great Lakes Basin, showed Raman bands potentially associated with carotenoids or chlorophyll.^47,48^ These interfering signals from lake water overwhelmed the signals from PS and PE NPs, leading to false positives in the Raman maps (Figure 4b,d). Oxidative digestion in heated hydrogen peroxide solution effectively removed these chemical interferences, producing clear dark-field images and clean Raman map backgrounds (Figures 4f and S19b). Notably, no PS or PE NPs were detected in the raw lake water samples from Lake Mendota and Lake Michigan when a 100 mL sample volume was used, implying that the NP concentrations at these sites were likely below the limit of detection (0.5 μg L^–1^). Following digestion, the Raman map processed with Pre_seg clearly differentiated and mapped PS and PE NPs on AAO membrane sensors (Figures 4h and S19d). These findings highlight the significance of removing organic matter from lake water for accurate NP detection. The slightly higher dissolved organic carbon (DOC) in Lake Mendota (4.4 mg L^–1^) in comparison to Lake Michigan (3.4 mg L^–1^), as well as potentail differences in DOC compositions (i.e., the aromaticity of the compounds), may make Lake Michigan more complicated for digestion. Despite these variations, oxidative digestion effectively removed chemical interferences from both sets of lakes. AAO membrane sensors remained structurally intact during the filtration step involving hydrogen peroxide. PS and PE NPs spiked in both lakes as a mixture were accurately distinguished (Figures 4h and S20), showing the capability of AAO membrane sensors coupled with Pre_seg for precise NP detection across diverse environmental samples.

**Figure 4:**
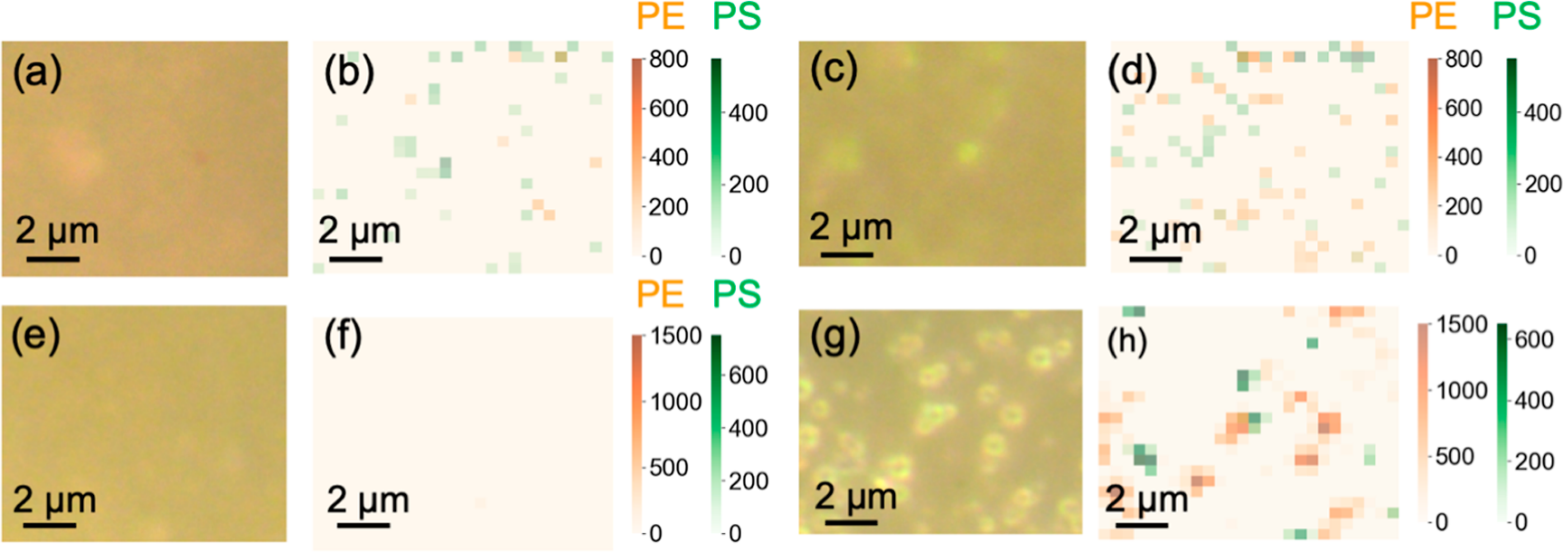
Influence of lake water matrices on PS and PE NP analysis using Pre_seg-assisted AAO membrane sensors. Dark-field images of 20 nm AAO membranes after filtering (a) raw and (e) digested lake water without spiked NPs. Corresponding Raman maps of the AAO membranes after (b) raw and (f) digested lake water filtration generated by tracking the intensities of PS Raman band at 996 cm^–1^ (green) and PE Raman band at 2872 cm^–1^ (orange). Dark-field images of AAO membranes after filtering raw lake water spiked with 500 nm PS NPs (c) without and (g) with digestion. Corresponding Raman maps of the AAO membranes after filtering raw lake water spiked with 500 nm PS NPs (d) without and (h) with digestion, generated by tracking the intensities of PS Raman band at 996 cm^–1^ (green) and PE Raman band at 2872 cm^–1^ (orange). All Raman maps were processed using Pre_seg.

## 4 Environmental Implications

Despite growing attention to NPs in recent decades, significant knowledge gaps persist regarding their occurrence and toxicity in aquatic systems, largely due to the lack of accurate analytical methods. In this study, we developed a Raman spectral processing algorithm, Pre_seg, paired with a regenerable AAO membrane sensor to enable simultaneous imaging, chemical characterization, and quantification of individual and mixed PS and PE NPs in real lake water. The commercial AAO membrane was transformed into a dual-function membrane sensor, acting as both a membrane filter for NP separation and a Raman substrate for in situ NP detection to minimize sample contamination and loss during transfer.

The Raman spectral processing algorithm, Pre_seg, integrated Raman band shift, intensity, and width, preserving the Raman spectral patterns of NPs while compressing spectra to fewer than 10 characteristic entries. Pre_seg achieved a prediction accuracy of ≥93% for PS NPs and improved non-NP rejection accuracy to ≥90.4% in real lake water datasets, which was 62.9% higher than Pre_fun that was developed previously in our lab. These capabilities enabled the generation of high-quality Raman maps of 500 and 300 nm PS NPs on the regenerable AAO membrane sensors, allowing accurate quantification regardless of NP size. The ultrasensitivity of this method outperforms existing methods based on standard Raman microscope and approaches environmentally relevant concentrations.^16,49,50^ The limit of detection can be further reduced to the 10^–3^ μg L^–1^ level by increasing the sample filtration volume to over 5 L. The membrane sensor’s rapid measurement capability, combined with Pre_seg and artificial intelligence algorithms, will potentially offer an automatic, real-time tool for the detection of NPs in environmental samples that are above or slightly below the diffraction limit of the Raman microscope. The AAO membrane sensors integrated with Pre_seg enabled the simultaneous morphological and chemical characterization, providing a powerful and fast tool to investigate the fundamental NP behaviors in complex matrices, such as aggregation mechanism of NPs in the freshwater environments. For NPs smaller than 200 nm, which are invisible under optical microscope, further studies are needed to develop a holistic detection strategy by coupling membrane sensors and Pre_seg with advanced imaging and data analytics tools.^51^

The importance of sample digestion in NP detection in real environmental water is highlighted in this study. Chemical interferences present in lake water can obscure dark-field images and mask Raman signals from NPs, complicating accurate detection.^14^ The adsorption of chemicals or the formation of biofilms on NP surfaces may alter their physicochemical properties, compromising the accuracy of Raman imaging and analysis.^52^ Oxidative digestion coupled with Pre_seg effectively cleaned up the samples, removed background noise and matrix interference, and enabled the accurate detection of PS and PE NPs spiked into two different Great Lake matrices. This approach also minimized overfitting during spectral matching while preserving essential Raman features of NPs, demonstrating the potential of deploying Pre_seg for detecting a broad range of NPs beyond PS and PE. Further studies are needed to optimize the digestion process to maximize NP recovery while minimizing damage to brittle NPs from various lake water matrices.^53^ Spherical PS and PE NPs were used in this study to demonstrate and validate the feasibility and reliability of Pre_seg and the AAO membrane sensors across various environmental matrices. Further work is needed to evaluate the detection performance of Pre_seg for nonspherical NPs such as fibers and fragments. The AAO membrane sensor achieved quantitative detection of individual and mixed NPs at a scanning rate of 0.1 s/pixel in both DI water and lake water, providing a low-cost and reproducible alternative to SERS substrates.^19,54^ This capability addresses the longstanding challenge of prolonged measurement time and limited accuracy in single-particle detection of NPs in environmental matrices.

## Supplementary Material



## Notes

Characterization of AAO membrane sensor. Calculation of prediction and rejection accuracy. SEM images of pristine 20 nm AAO membranes. Optical images and Raman maps of 500 and 300 nm PS of four concentrations on 20 nm AAO membranes. Raman spectra collected from pristine AAO membranes. Selected individual Raman spectra of pristine AAO membranes. Examples of the processed Raman spectra of individual 500 nm PS NPs on AAO membranes and pristine AAO membranes. Raman spectra of individual 500 and 300 nm PS NPs on AAO membranes before and after baseline correction. Calculated FWHM of five characteristic Raman bands of PE NPs. Raman spectra of PVC, PP, PS, and PE MPs acquired from the spectral library of plastic particles (SLoPP). Randomly selected 12 Raman spectra collected from the AAO membranes after raw lake water filtration after Pre_fun processing. Dark-field images and Raman maps of 500 and 300 nm PS NPs on 20 nm AAO membranes after each step of Pre_seg processing. Calibration curves of (a) 500 and (b) 300 nm PS NPs on 20 nm AAO membranes generated by Raman maps processed only with baseline correction. Dark-field images and Raman maps of mixed 500 and 300 nm PS on 20 nm AAO membranes. Generalized calibration curves of 500 and 300 nm PS NPs on 20 nm AAO membranes processed only with baseline correction. Dark-field image and the corresponding Raman map of the AAO membrane processed with Pre_seg after one regeneration cycle. A collection of Raman spectra from the Raman maps in Figure 4. Dark-field image and Raman map of PS and PE spiked in Lake Michigan water on a 20 nm AAO membrane. Dark-field images of the regenerated AAO membrane sensor after one cycle of traditional cleaning (PDF)
