# Supplementary material for: Regenerable Membrane Sensors for Ultrasensitive Nanoplastic Quantification Enabled by A Data-driven Raman Spectral Processing Algorithm
Source: Environ Sci Technol. 2025 Jul 29;59(31):16652–61. doi: 10.1021/acs.est.5c05396 (PMC12503354; doi:10.1021/acs.est.5c05396)
Supplement: Supplementary file 1 [file es5c05396_si_001.pdf]

Supporting Information for

Regenerable Membrane Sensors for Ultrasensitive Nanoplastic Quantification Enabled by A Data-driven Raman Spectral Processing Algorithm

Ziyan Wu[1], Sarah E. Janssen[2], Michael T. Tate[2], Mohan Qin[1], Haoran Wei[1,3*]*

1. *Department of Civil and Environmental Engineering, University of Wisconsin–Madison, Madison, Wisconsin 53706, USA*
2. *U.S. Geological Survey, Upper Midwest Water Science Center, Madison, Wisconsin 53726, USA*
3. *Environmental Chemistry and Technology Program, University of Wisconsin–Madison, Madison, Wisconsin 53706, USA*

* Corresponding author. E-mail: mohan.qin@wisc.edu; haoran.wei3@wisc.edu

Number of pages: 24

Number of figures: 20

Number of texts: 2

Any use of trade, firm, or product names is for descriptive purposes only and does not imply endorsement by the U.S. Government.

Supplementary Text 1. Characterization of AAO membrane sensor.

Anodic aluminum oxide (AAO) membranes were shown to be hydrophilic, evidenced by the small contact angle (21.5°) between a deionized (DI) water drop and the AAO membrane surface (Figure 1a). The hydrophilicity of the AAO membrane benefited the nanoplastic (NP) separation from water samples with shorter filtration time due to the potential higher water permeability compared to more hydrophobic membranes.[1] Scanning electron microscope (SEM) images of AAO membranes demonstrated the porous and flat surface structure of AAO membrane (Figure 1b-c, Figure S1). Such surface structure was proven to be beneficial for low-micrometer microplastic and pathogen imaging in previous studies.[2,3]

Supplementary Text 2. Calculation of prediction and rejection accuracy.

The calculation of prediction accuracy was performed on the Raman dataset of individual polystyrene (PS) spectra. The calculation of rejection accuracy was performed on three Raman datasets generated in this study, including pristine AAO membranes, AAO membranes after raw lake water filtration, and AAO membranes after digested lake water filtration. The Raman spectra from each dataset were processed with Pre_fun and Pre_seg. The processed spectra with Pre_fun and the individual steps of Pre_seg were output and saved for the accuracy calculation. For the processed spectra in each dataset, the non-zero entries of one PS Raman band (996 cm^{-1}) were recorded. The prediction accuracy was calculated as:

$$Predicting\ accuracy = \frac{Number\ of\ non-zero\ entries\ of\ the\ tracked\ peak}{Number\ of\ spectra\ in\ a\ dataset} \times 100\%$$

The rejection accuracy was calculated as:

$$Rejecting\ accuracy = 1 - \frac{Number\ of\ non-zero\ entries\ of\ the\ tracked\ peak}{Number\ of\ spectra\ in\ a\ dataset} \times 100\%$$

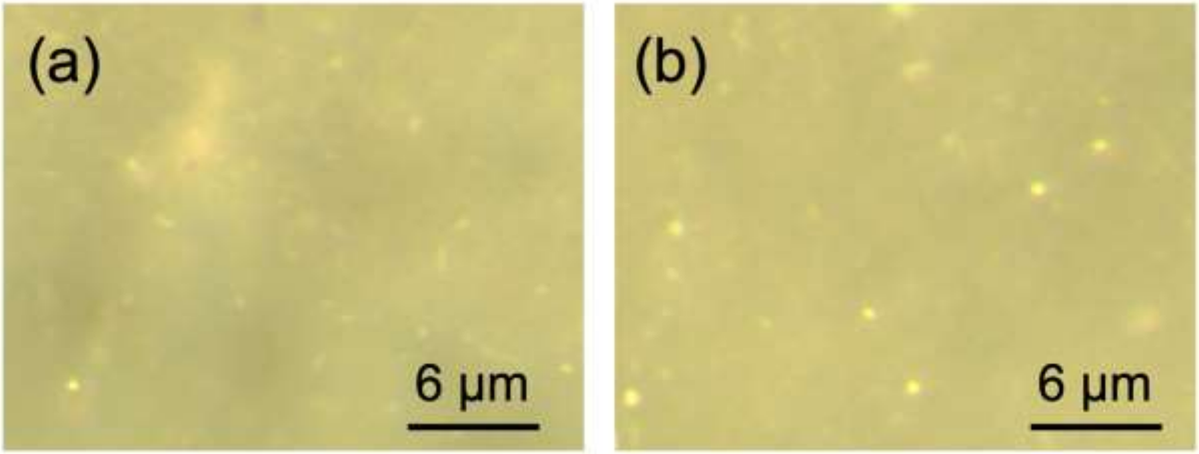

Supplementary Fig. 1. Dark-field images of two randomly selected locations on the regenerated 200 nm anodic aluminum oxide (AAO) membrane sensor after one cycle of traditional cleaning. One cycle of cleaning includes 50 mL deionized water backwashing, followed by 50 mL 10% bleach washing, and another 50 mL deionized water backwashing.

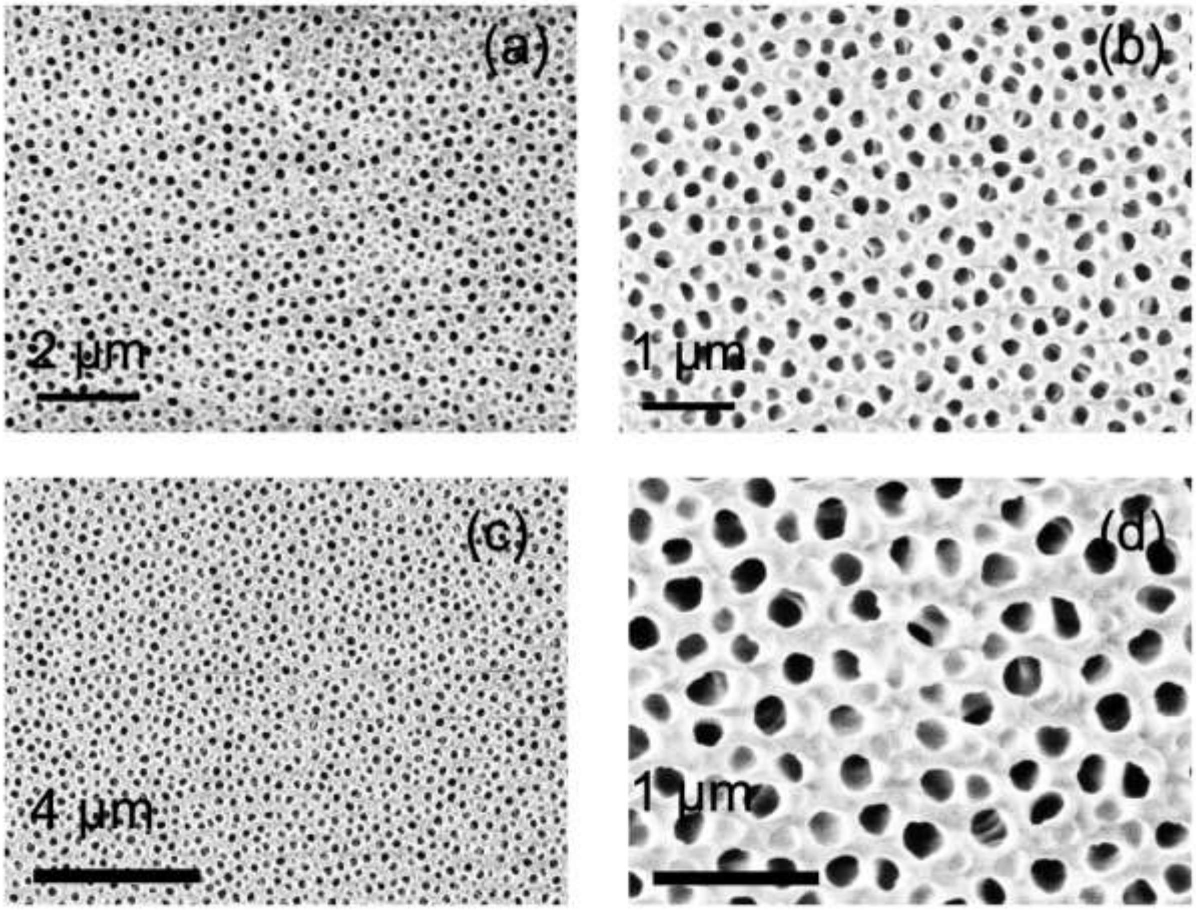

Supplementary Fig. 2. Scanning electron microscope (SEM) images of pristine 200 nm anodic aluminum oxide (AAO) membranes.

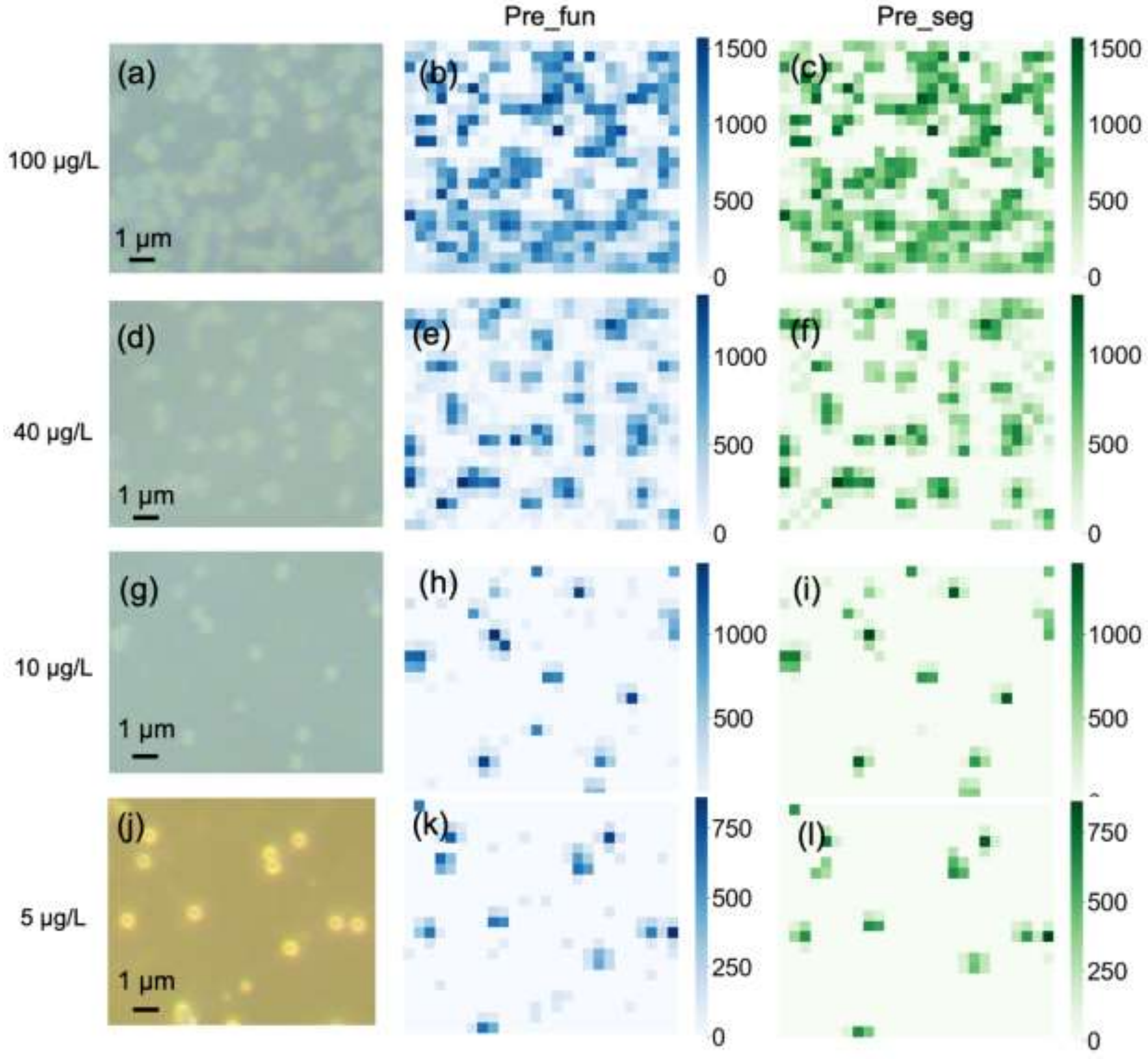

Supplementary Fig. 3. Optical images and Raman maps of 500 nm polystyrene (PS) nanoplastics (NPs) of four concentrations (i.e., 100, 40, 10, and 5 μg/L) on 20 nm anodic aluminum oxide (AAO) membranes. Color gradients represent detection intensity in arbitrary units (AU). Raman maps in (b), (e), (h), and (k) were processed with Pre_fun. Raman maps in (c), (f), (i), and (l) were processed with Pre_seg. Raman maps were generated by tracking the intensity of the PS Raman band at 996 cm^{-1}. The spiked concentrations were calculated based on the known stock concentrations and the corresponding dilution factors.

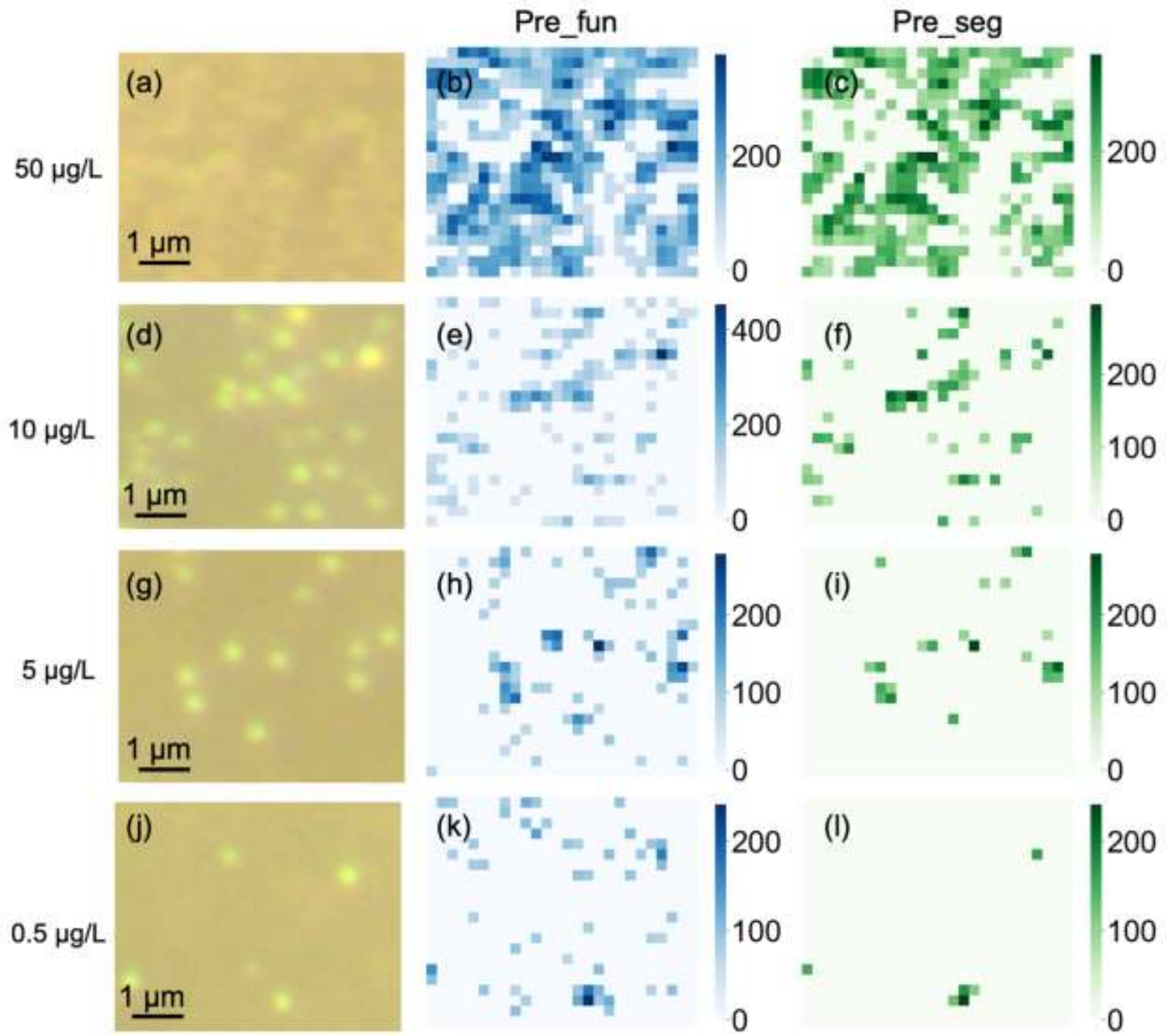

Supplementary Fig. 4. Optical images and Raman maps of 300 nm polystyrene (PS) nanoplastics (NPs) of four concentrations (i.e., 50, 10, 5, and 0.5 μg/L) on 20 nm anodic aluminum oxide (AAO) membranes. Color gradients represent detection intensity in arbitrary units (AU). Raman maps in (b), (e), (h), and (k) were processed with Pre_fun. Raman maps in (c), (f), (i), and (l) were processed with Pre_seg. Raman maps were generated by tracking the intensity of the PS Raman band at 996 cm^{-1}. The spiked concentrations were calculated based on the known stock concentrations and the corresponding dilution factors.

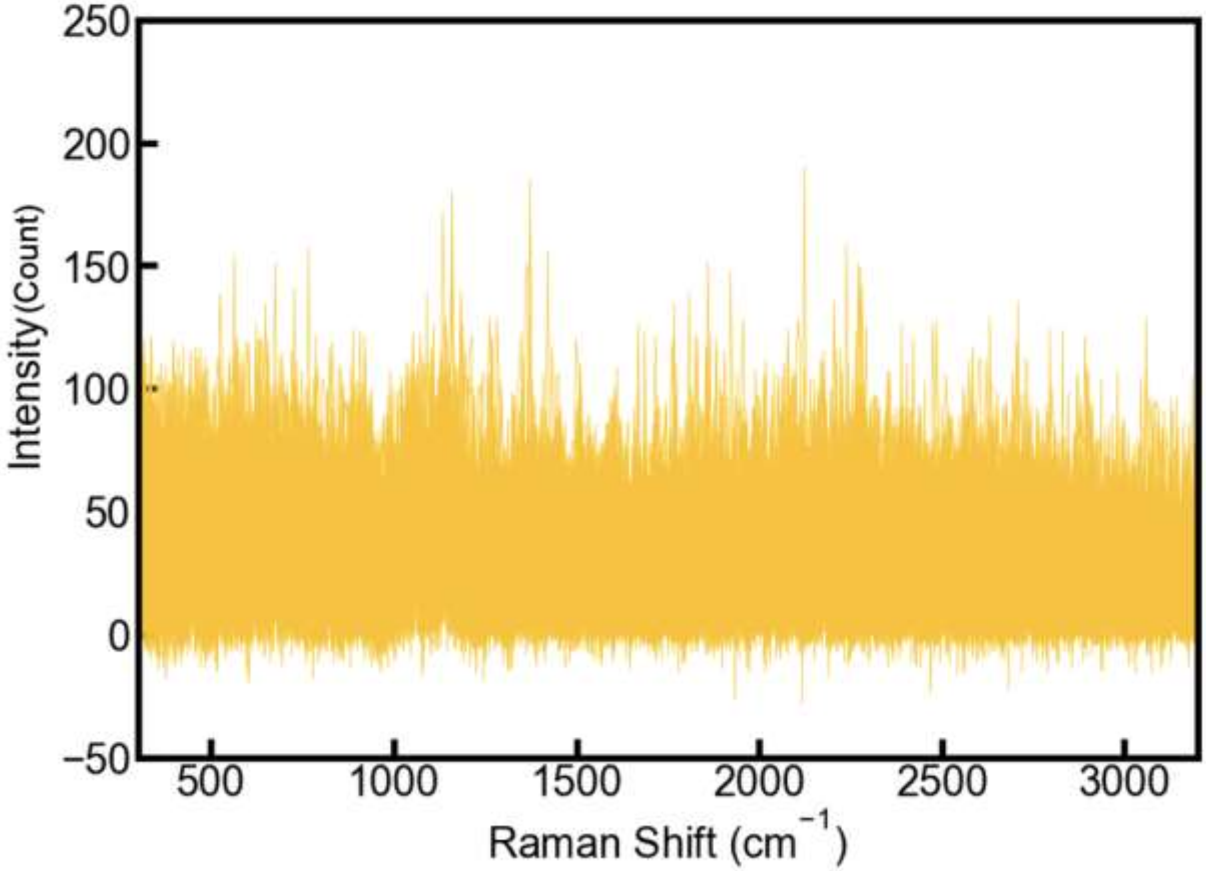

Supplementary Fig. 5. A collection of 572 Raman spectra collected from pristine anodic aluminum oxide (AAO) membranes.

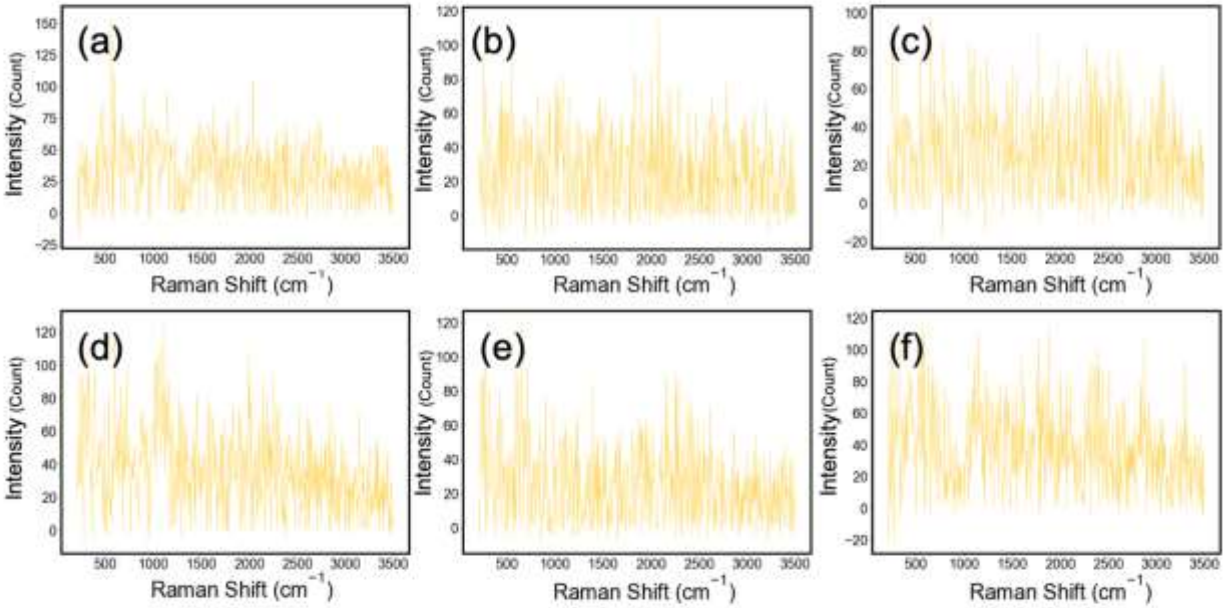

Supplementary Fig. 6. Six individual Raman spectra collected from of pristine anodic aluminum oxide (AAO) membranes randomly selected from the 572 spectra shown in Fig. S5.

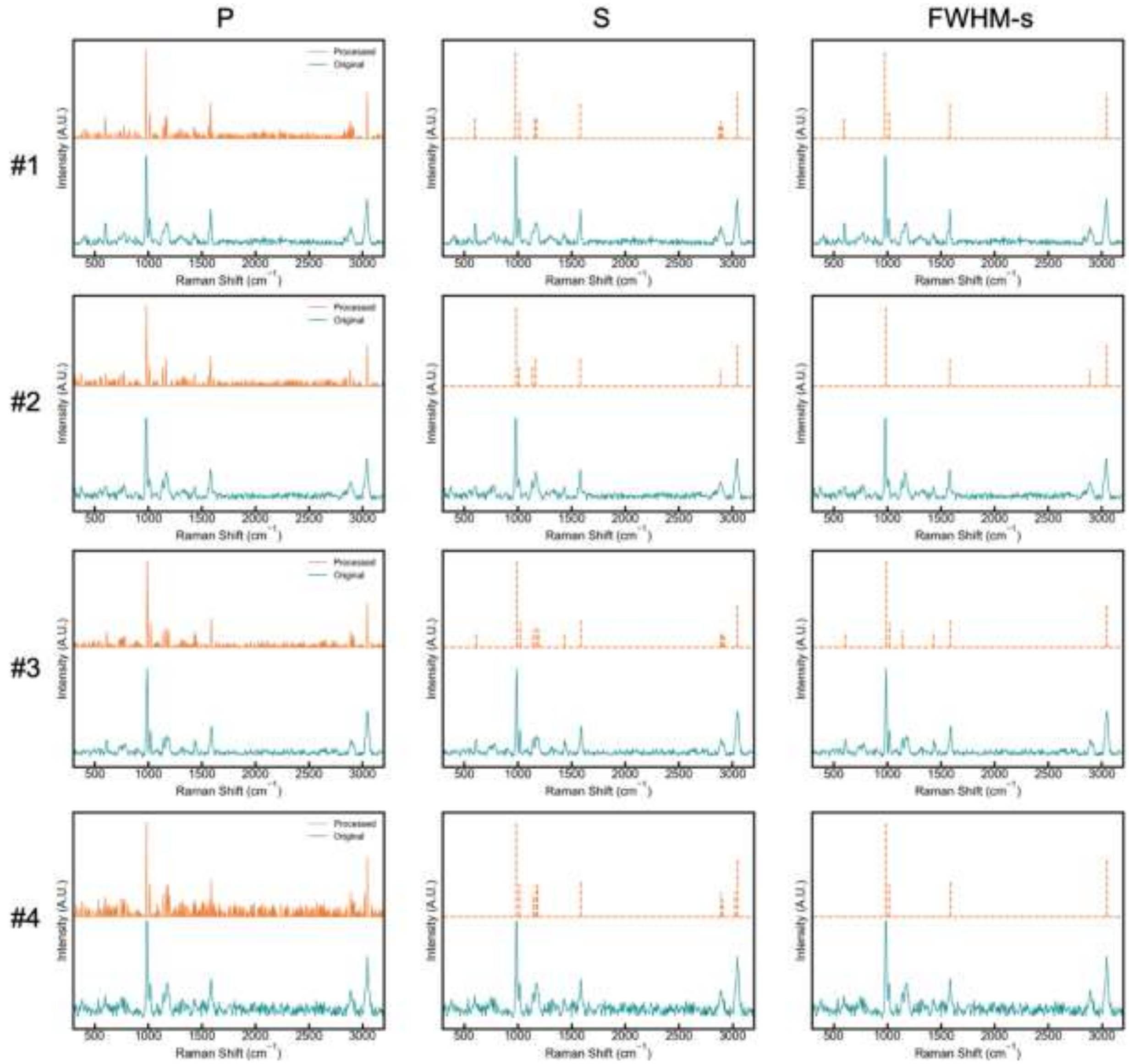

Supplementary Fig. 7. Four examples of the processed Raman spectra of individual 500 nm polystyrene (PS) nanoplastics (NPs) on anodic aluminum oxide (AAO) membranes after peak detection (P, first column), denoising based on signal to noise ratios SNR (S, second column), and denoising based on full width at half maximums (FWHMs) (FWHM-s, third column) steps in Pre_seg. Despite variations in the number of retained peaks, the overall Raman fingerprint of PS NPs remained consistent in terms of peak positions, intensities, and relative rankings, highlighting the robustness of Pre_seg in processing PS NP spectra.

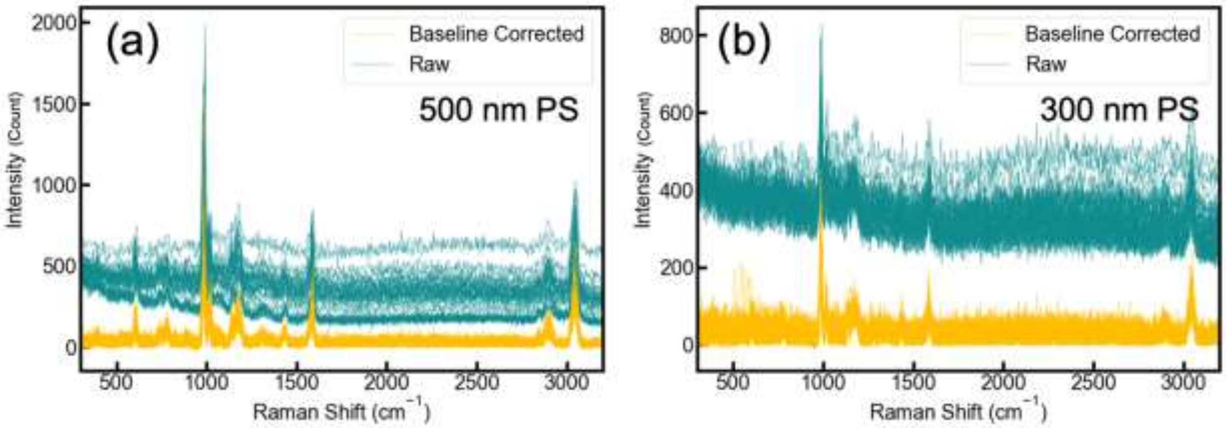

Supplementary Fig. 8. Raman spectra of individual 500 and 300 nm polystyrene (PS) nanoplastics (NPs) on anodic aluminum oxide (AAO) membranes before (cyan) and after (yellow) baseline correction.

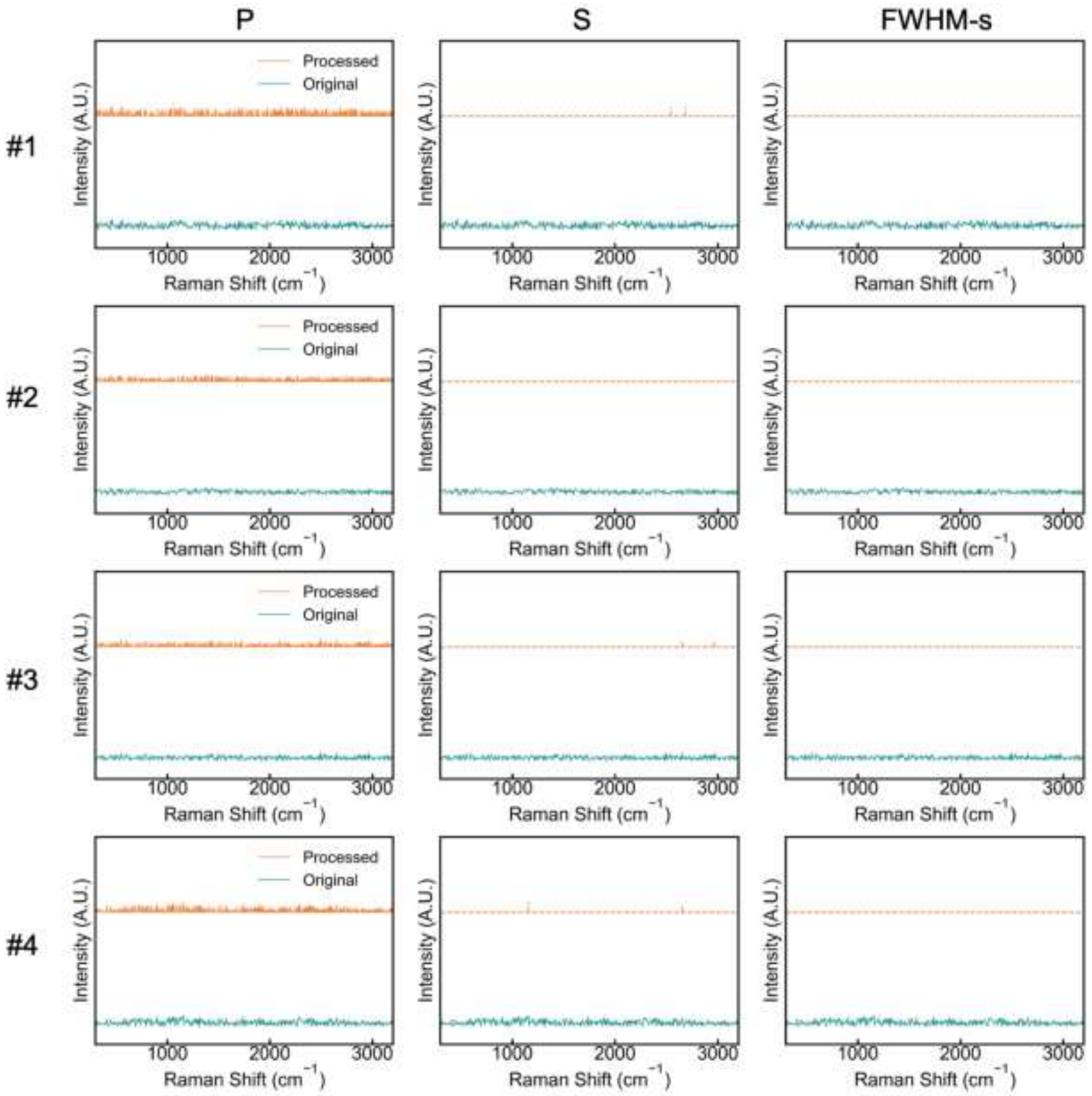

Supplementary Fig. 9. Four examples of the processed Raman spectra of pristine anodic aluminum oxide (AAO) membranes after peak detection (P, first column), denoising based on signal to noise ration (SNR) (S, second column), and denoising based on full width at half maximums FWHMs (FWHM-s, third column) steps in Pre_seg.

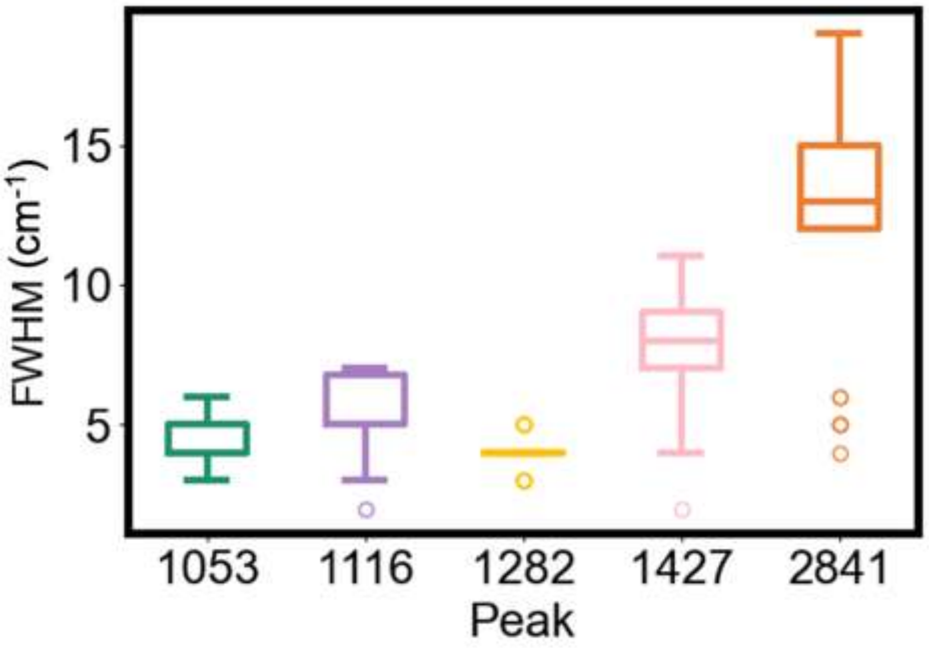

Supplementary Fig. 10. Calculated full width at half maximums (FWHM) in cm^{-1} of five characteristic Raman bands of polyethylene (PE) nanoplastics (NPs).

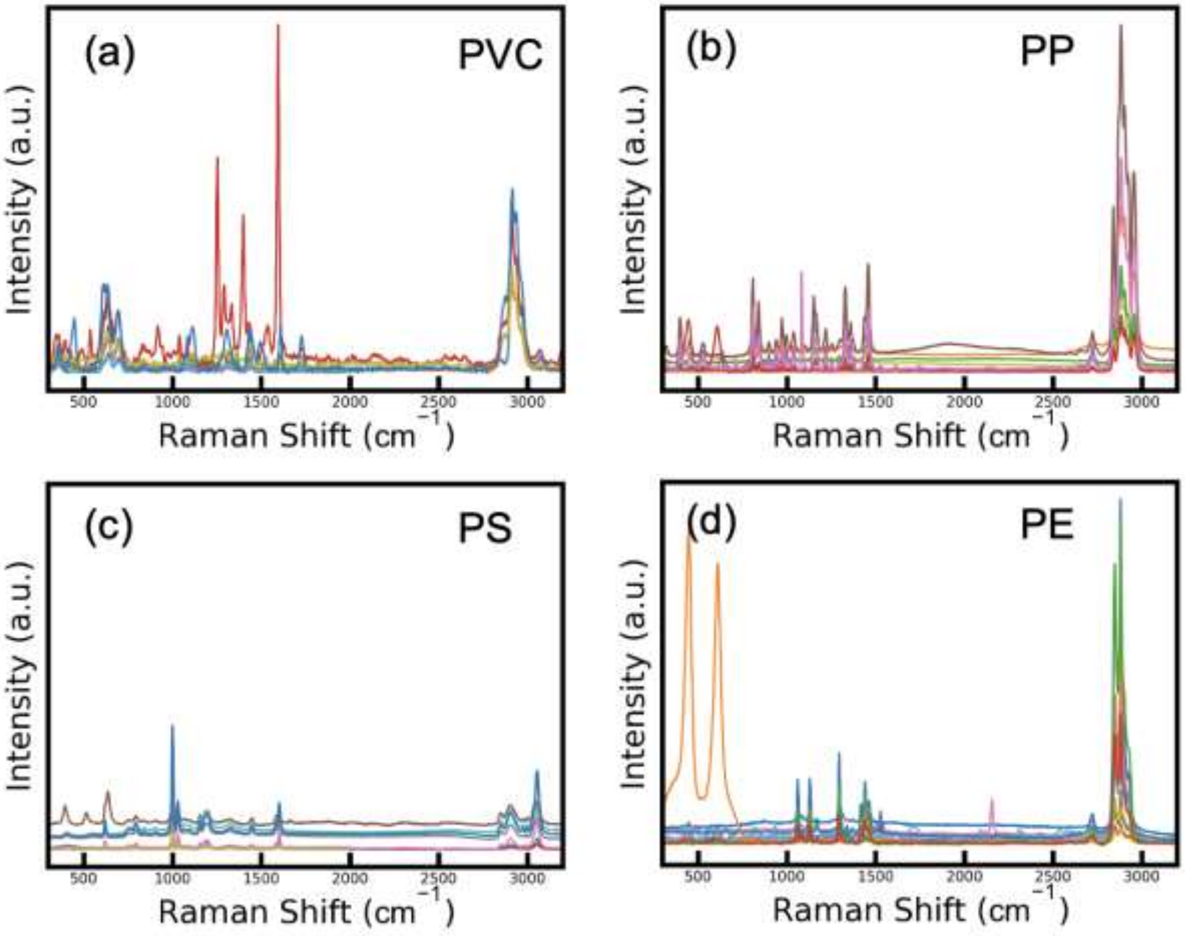

Supplementary Fig. 11. Raman spectra of polyvinyl chloride (PVC), polypropylene (PP), polystyrene (PS), and polyethylene (PE) microplastics (MPs) acquired from the spectral library of microplastics aged in the environment (termed SLoPP).[4] Each line represents one type of microplastics from the library.

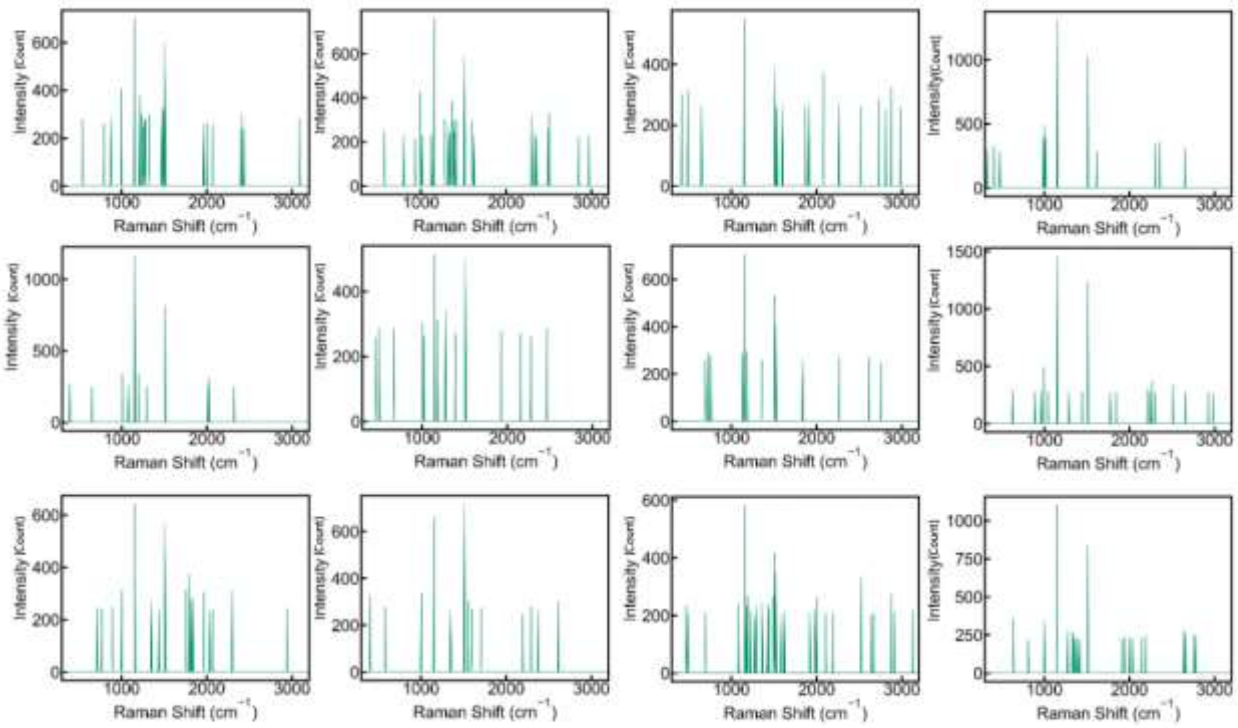

Supplementary Fig. 12. Randomly selected twelve Raman spectra collected from the anodic aluminum oxide (AAO) membranes after raw lake water filtration after Pre_fun processing.

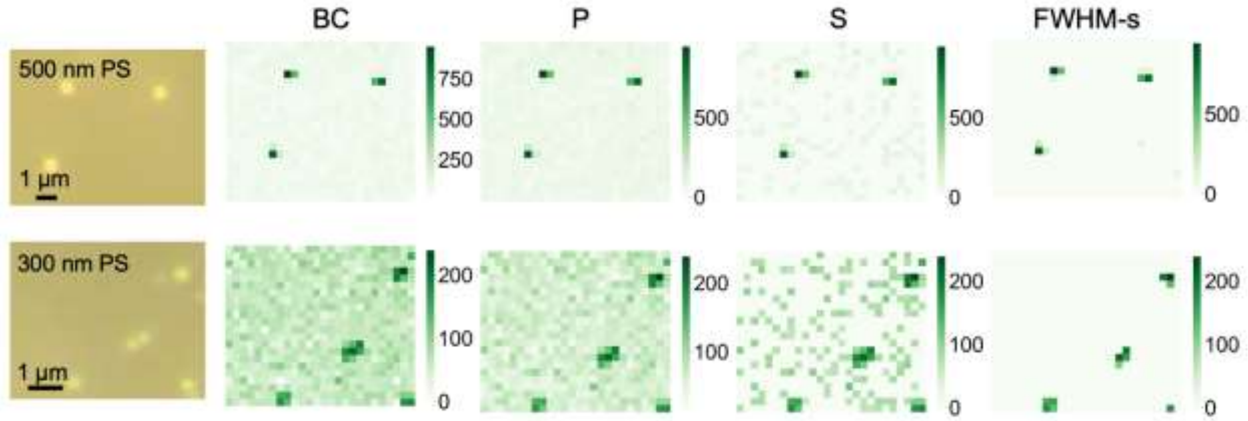

Supplementary Fig. 13. Dark-field images and Raman maps of 500 and 300 nm polystyrene (PS) nanoplastics (NPs) on 20 nm anodic aluminum oxide (AAO) membranes after each step of Pre_seg processing. Raman maps were generated by tracking the intensity of the PS Raman band at 996 cm^{-1}. Concentrations of 500 and 300 nm PS were both 1 μg/L. Color gradients represent detection intensity in arbitrary units (AU).

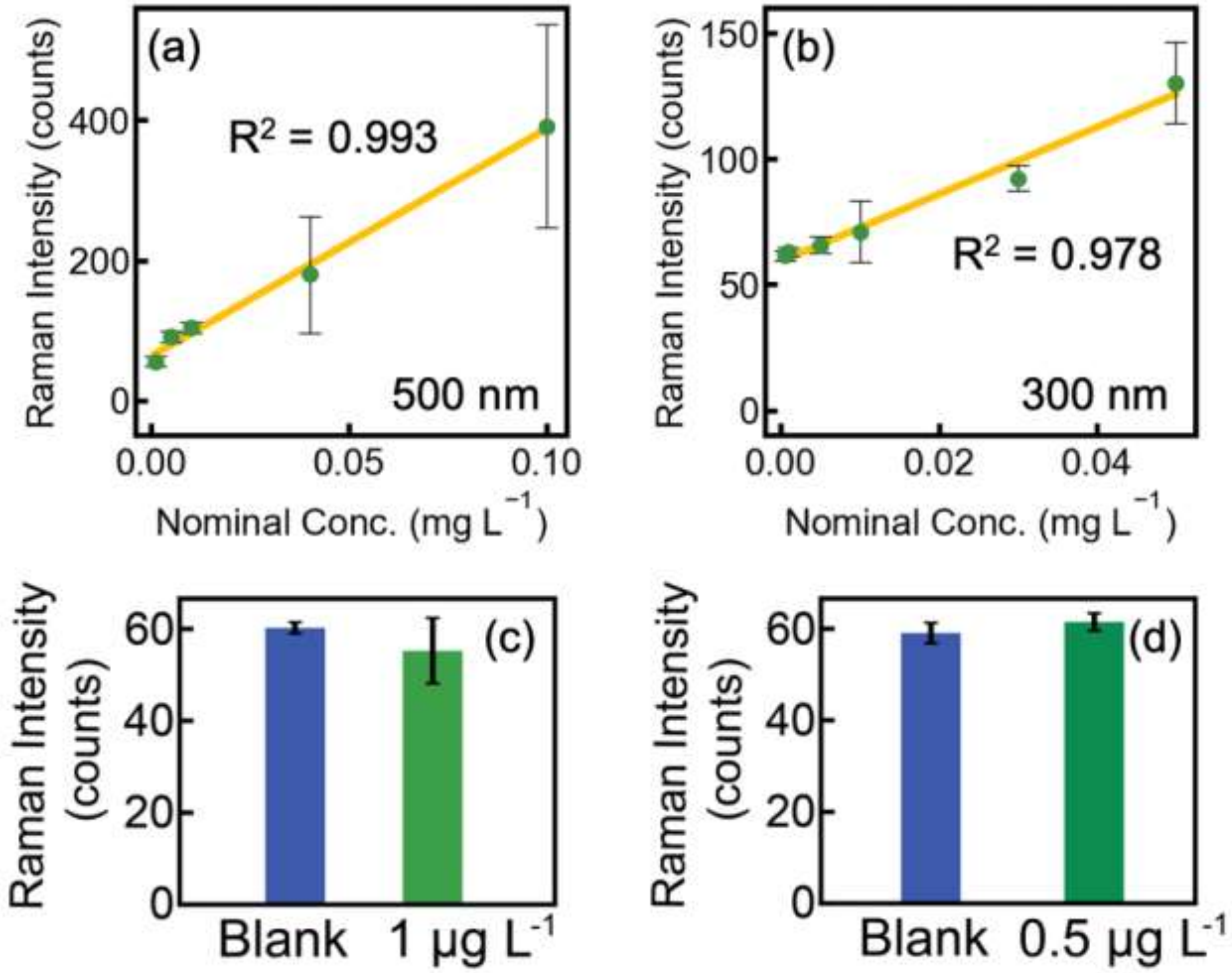

Supplementary Fig. 14. Calibration curves of (a) 500 and (b) 300 nm PS NPs on 20 nm AAO membranes generated by Raman maps processed only with baseline correction. (c) Averaged Raman intensities of Raman maps tracking the 996 cm□¹ band for a blank sample (blue, left bar) and a sample containing 1 μg/L of 500 nm PS NPs (green, right bar) on 20 nm AAO membranes. (d) Averaged Raman intensities of Raman maps tracking the 996 cm□¹ band for a blank sample (blue, left bar) and a sample containing 0.5 μg/L of 300 nm PS NPs (green, right bar) on 20 nm AAO membranes.

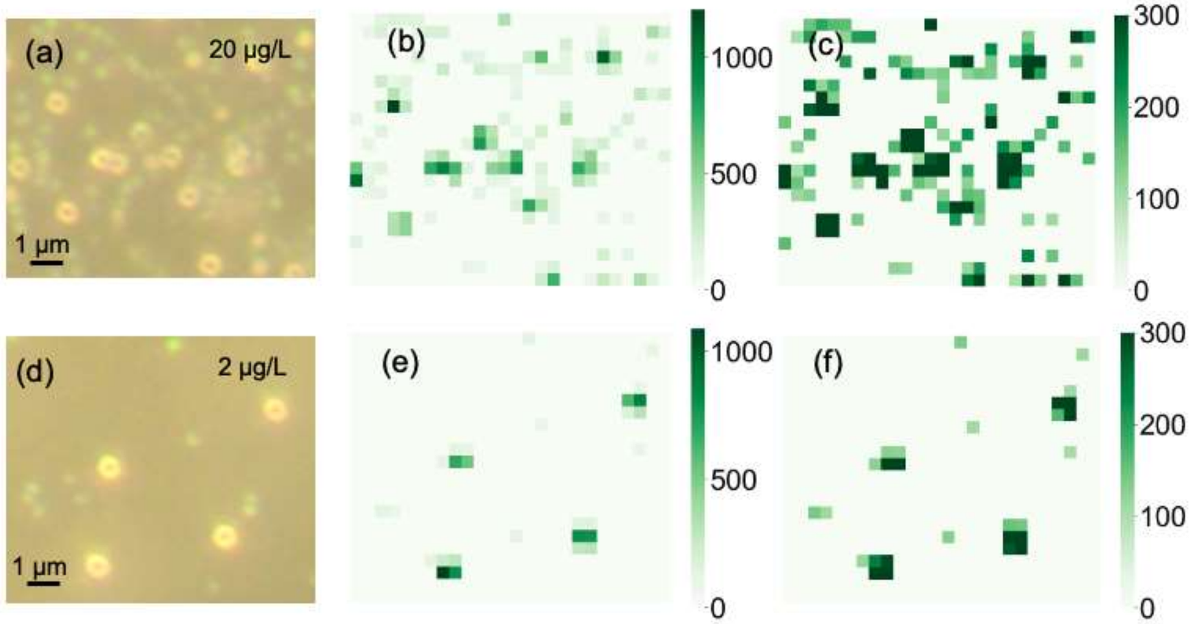

Supplementary Fig. 15. Dark-field images and Raman maps of mixed 500 and 300 nm polystyrene (PS) on 20 nm anodic aluminum oxide (AAO) membranes. Color gradients represent detection intensity in arbitrary units (AU). Dark-field images of mixed 500 and 300 nm PS with a total concentration of (a) 20 and (d) 2 μg/L. The suspensions were prepared with an equal mass proportion of 500 and 300 nm particles. (b) Raman map generated with Pre_seg for the imaging area in (a). (c) The scale of color bar was changed from 0 – 1200 in (b) to 0 – 300. (e) Raman map generated with Pre_seg for the imaging area in (d). (f) The scale of color bar was changed from 0 – 1200 in (e) to 0 – 300. Raman maps were generated by tracking the intensity of the PS Raman band at 996 cm^{-1}. The spatial resolution of the maps was 500 nm. Without re-scaling the color bar, 500 nm nanoplastics (NPs) were much more visible than the 300 nm NPs in the Raman maps, matching with the imaging result of NPs in the dark-field images. The visualization of 300 nm NPs was improved when the color bar was re-scaled to the Raman intensity of 300 nm NPs, demonstrating the potential of using Raman maps processed with Pre_seg as a reliable tool for precisely imaging mixed-size NPs.

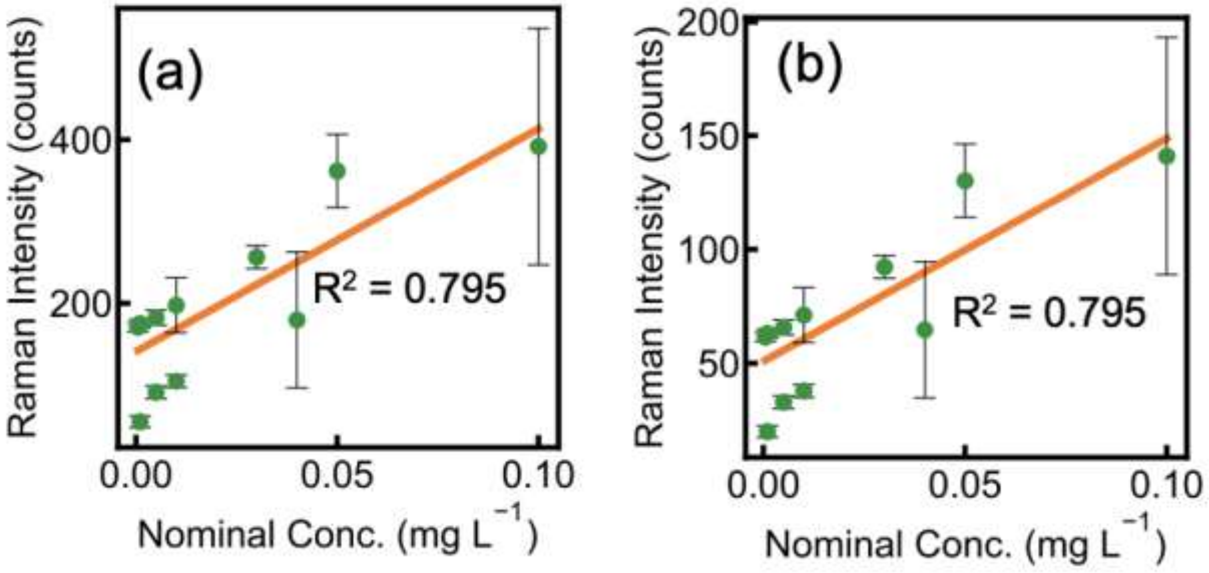

Supplementary Fig. 16. Generalized calibration curves of 500 and 300 nm polystyrene (PS) nanoplastics (NPs) on 20 nm anodic aluminum oxide (AAO) membranes with a mapping spatial resolution of (a) 500 and (b) 300 nm. The calibration curves were generated by Raman maps processed only with baseline correction.

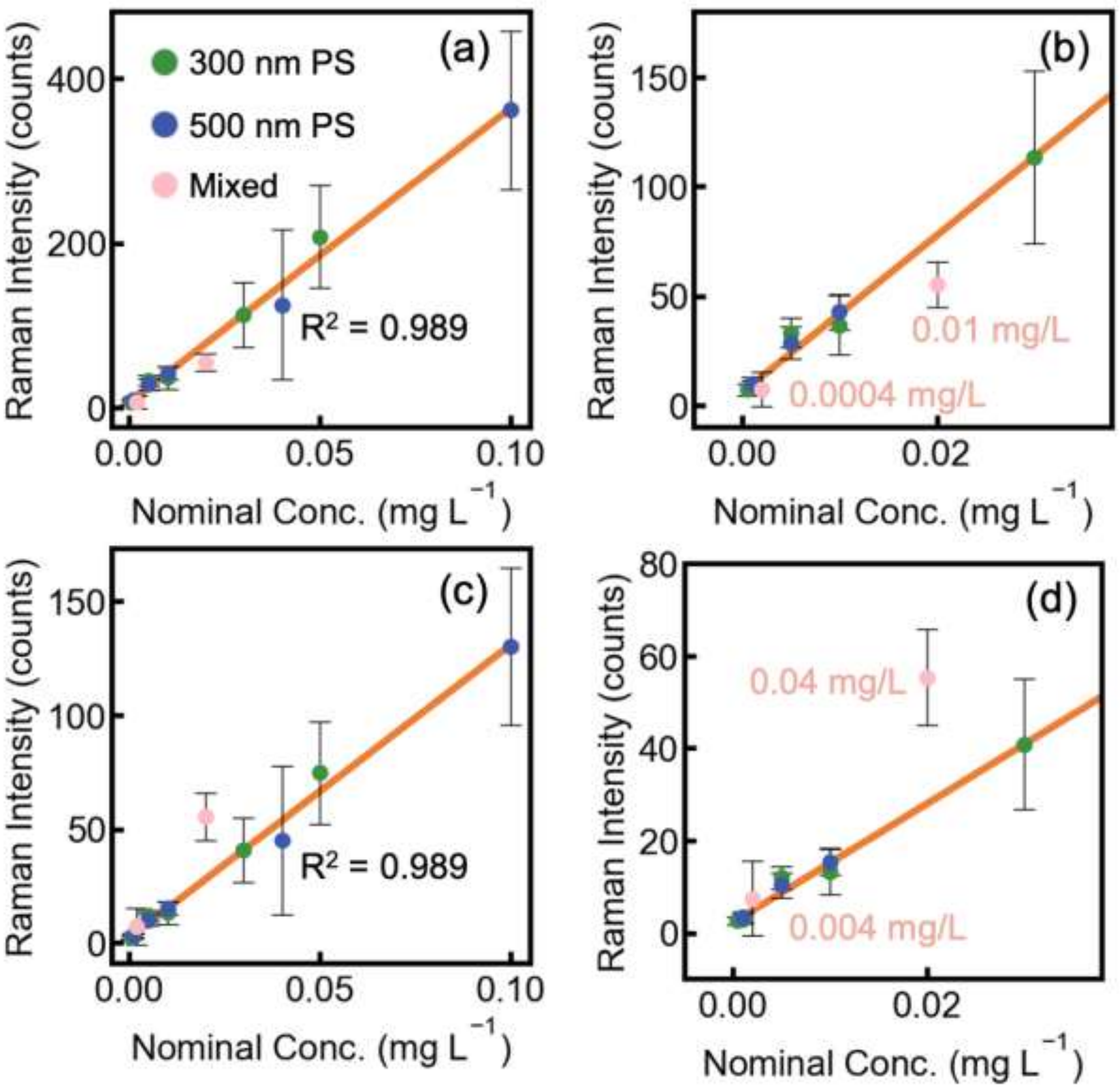

Supplementary Fig. 17. Generalized calibration curves for 500 and 300 nm polystyrene (PS) nanoplastics (NPs) on 20 nm anodic aluminum oxide (AAO) membranes with a mapping spatial resolution of (a) 500 and (c) 300 nm. (b) Zoomed-in plot of (a). (d) Zoomed-in plot of (c). The calibration curves were generated by Raman maps processed by Pre_seg.

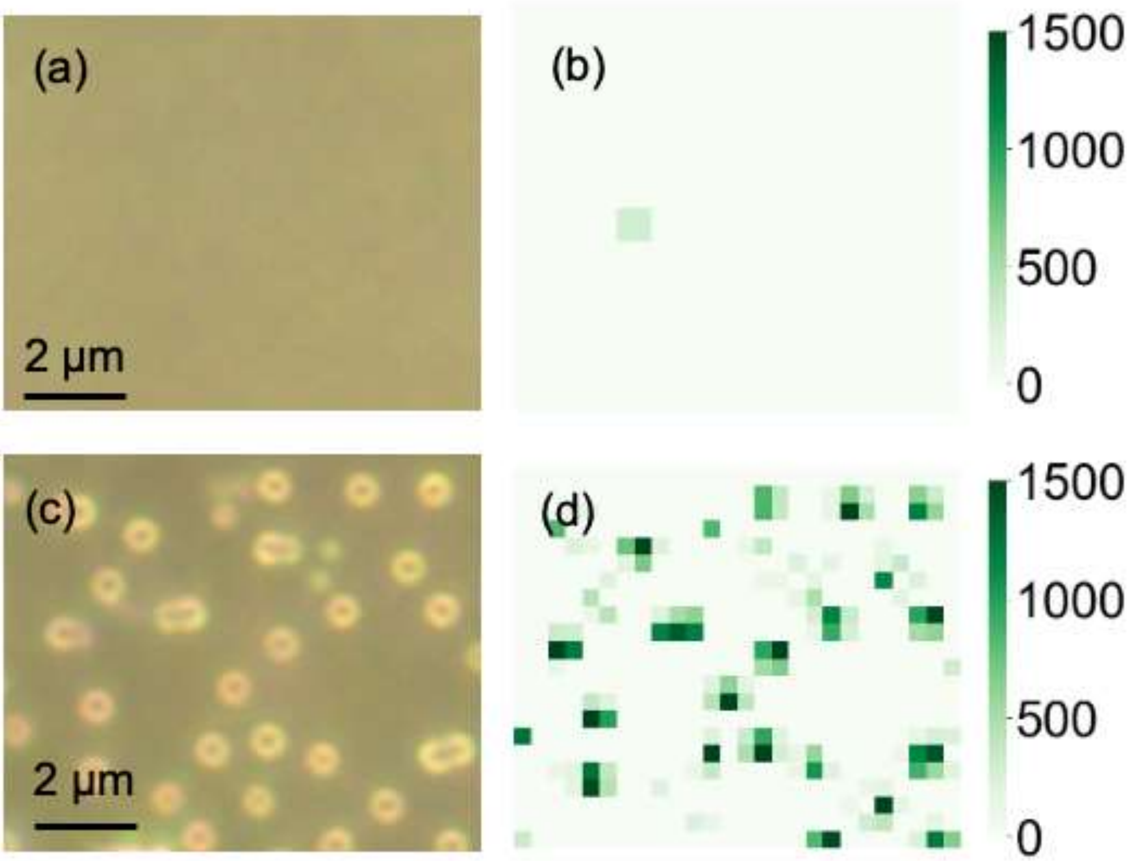

Supplementary Fig. 18. (a) Dark-field image of anodic aluminum oxide (AAO) membrane after one regeneration cycle. (b) The corresponding Raman map of the AAO membrane processed with Pre_seg. (c) Dark-field image of 500 nm polystyrene (PS) nanoplastics (NPs) on AAO membrane after one regeneration and reuse cycle. (d) The corresponding Raman map of the reused AAO membrane processed with Pre_seg. Raman maps were generated by tracking the intensity of the PS Raman band at 996 cm^{-1}. Color gradients represent detection intensity in arbitrary units (AU).

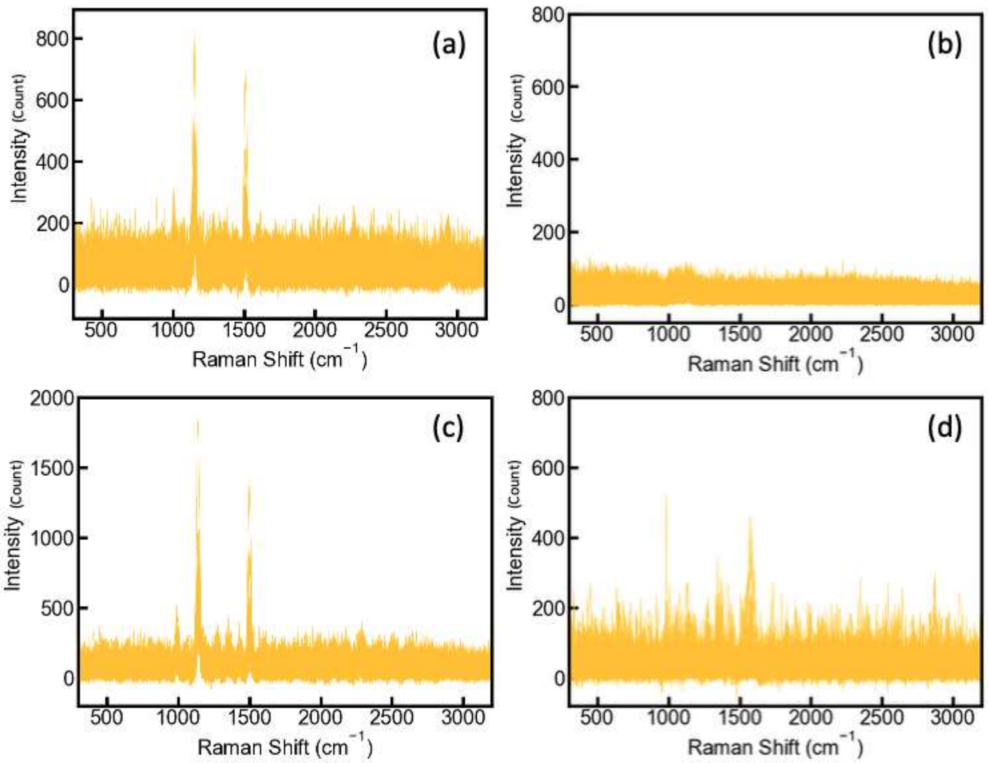

Supplementary Fig. 19. A collection of Raman spectra from the Raman maps in Figure 4. (a) Raman spectra of the Raman map in Figure 4b after baseline correction, corresponding to the Lake Mendota water sample without spiked polystyrene (PS) and polyethylene (PE) nanoplastics (NPs) and without sample digestion. (b) Raman spectra of the Raman map in Figure 4f after baseline correction, corresponding to the Lake Mendota (Madison, Wisconsin, USA) water sample without spiked PS and PE NPs and with sample digestion. (c) Raman spectra of the Raman map in Figure 4d after baseline correction, corresponding to the Lake Mendota water sample spiked with PS and PE NPs and without sample digestion. (d) Raman spectra of the Raman map in Figure 4h after baseline correction, corresponding to the Lake Mendota water sample spiked with PS and PE NPs and with sample digestion.

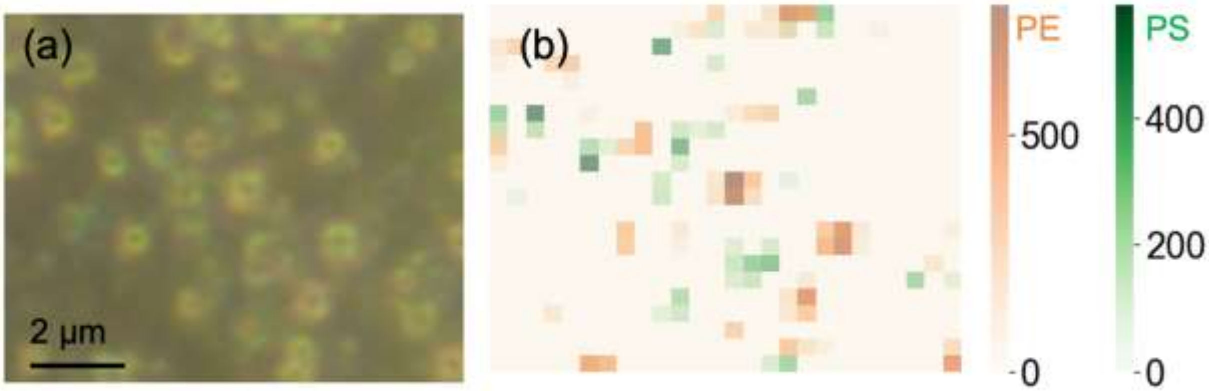

Supplementary Fig. 20. Dark-field image and Raman map of a 20 nm anodic aluminum oxide (AAO) membrane after filtering Lake Michigan (Laurentian Great Lakes, USA) water spiked with polystyrene (PS) and polyethylene (PE) nanoplastics (NPs) after sample digestion. The Raman map was processed with Pre_seg and generated by tracking the intensities of the PS Raman band at 996 cm^{-1} and the PE Raman band at 2872 cm^{-1}. Color gradients represent detection intensity in arbitrary units (AU).

Reference

(1) Wang, X.; Chen, X.; Yoon, K.; Fang, D.; Hsiao, B. S.; Chu, B. High Flux Filtration Medium Based on Nanofibrous Substrate with Hydrophilic Nanocomposite Coating. *Environ. Sci. Technol.* **2005**, *39* (19), 7684–7691. https://doi.org/10.1021/es050512j.

(2) Wigginton, K. R.; Vikesland, P. J. Gold-Coated Polycarbonate Membrane Filter for Pathogen Concentration and SERS-Based Detection. *Analyst* **2010**, *135* (6), 1320–1326. https://doi.org/10.1039/B919270K.

(3) Wu, Z.; Janssen, S. E.; Tate, M. T.; Wei, H.; Qin, M. Adaptable Plasmonic Membrane Sensors for Fast and Reliable Detection of Trace Low-Micrometer Microplastics in Lake Water. *Environ. Sci. Technol.* **2024**, *58* (45), 20172–20180. https://doi.org/10.1021/acs.est.4c06503.

(4) Munno, K.; De Frond, H.; O'Donnell, B.; Rochman, C. M. Increasing the Accessibility for Characterizing Microplastics: Introducing New Application-Based and Spectral Libraries of Plastic Particles (SLoPP and SLoPP-E). *Anal. Chem.* **2020**, *92* (3), 2443–2451. https://doi.org/10.1021/acs.analchem.9b03626.
